# Vitamin D in Neurological Diseases

**DOI:** 10.3390/ijms24010087

**Published:** 2022-12-21

**Authors:** Domenico Plantone, Guido Primiano, Carlo Manco, Sara Locci, Serenella Servidei, Nicola De Stefano

**Affiliations:** 1 Centre for Precision and Translational Medicine, Department of Medicine, Surgery and Neuroscience, University of Siena, 53100 Siena, Italy; 2 Fondazione Policlinico Universitario A. Gemelli IRCCS, 00168 Rome, Italy; 3 Dipartimento Universitario di Neuroscienze, Università Cattolica del Sacro Cuore, 00168 Rome, Italy

**Keywords:** vitamin D, neurodegeneration, inflammation, gene transcription

## Abstract

Vitamin D may have multiple effects on the nervous system and its deficiency can represent a possible risk factor for the development of many neurological diseases. Recent studies are also trying to clarify the different effects of vitamin D supplementation over the course of progressive neurological diseases. In this narrative review, we summarise vitamin D chemistry, metabolism, mechanisms of action, and the recommended daily intake. The role of vitamin D on gene transcription and the immune response is also reviewed. Finally, we discuss the scientific evidence that links low 25-hydroxyvitamin D concentrations to the onset and progression of severe neurological diseases, such as multiple sclerosis, Parkinson’s disease, Alzheimer’s disease, migraine, diabetic neuropathy and amyotrophic lateral sclerosis. Completed and ongoing clinical trials on vitamin D supplementation in neurological diseases are listed.

## 1. Introduction

The multiple and complex effects of vitamin D on human health are more and more clear. Historically, the best-known effects have been those on bone, albeit many studies highlighted the so-called “non-calcemic actions” of vitamin D [1]. Increasing scientific attention has been paid to the association between vitamin D and neurological diseases [2,3]. There is a significant concern about the possibility that having vitamin D insufficiency could negatively influence the development of neurodegenerative and neuroinflammatory diseases [4]. From this perspective, vitamin D receptors are expressed in both neurons and glial cells in numerous important brain areas including substantia nigra, hippocampus, hypothalamus, thalamus, and subcortical grey nuclei [5]. In these regions, vitamin D seems to have a role in the differentiation and maturation of neurons, in the regulation of growth factors synthesis, including neural growth factor, and glial cell line-derived growth factor, and in the synthesis of different neurotransmitters including acetylcholine, dopamine, and gamma-aminobutyric acid [5].

This narrative review aims to summarise the biochemical and metabolic effects of vitamin D and the scientific evidence that links vitamin D insufficiency to the onset and progression of the main neurological diseases, such as multiple sclerosis (MS), Parkinson’s disease (PD), Alzheimer’s disease (AD), migraine, amyotrophic lateral sclerosis (ALS) and diabetic neuropathy.

## 2. Methods of Literature Search

To select the relevant literature for this narrative review the authors firstly searched PubMed, Embase and google scholar databases using the following string: “Vitamin D” OR “25-hydroxyvitamin D”, OR “vitamin D2”, OR “vitamin D3”, OR “ergosterol”, OR “cholecalciferol” OR “calcitriol “AND (“neurology” OR “nervous system” OR “brain” OR “neuropathology” OR “headache” OR “migraine” OR “immunity” OR “inflammation” OR “amyotrophic lateral sclerosis” OR ““motor neurone disease” OR “Lou Gehrig’s disease” OR “dementia” OR “Alzheimer’s disease” OR “multiple sclerosis” OR “demyelination” OR “Parkinson’s disease” OR “diabetic neuropathy”). Articles in languages other than English, published before the 1980s, or reported only as abstracts were not considered suitable and were discarded unless considered relevant. The authors then evaluated the abstracts of all articles and selected those focused on the aim of the present narrative review. The reference lists of all selected articles were also evaluated to identify additional relevant articles. The authors then critically reviewed the selected articles to remove those of low quality based on the number of enrolled subjects, the clinical relevance of the presented data, the robustness of the methods, and those works including redundant information.

## 3. Vitamin D Chemistry, Metabolism, Mechanisms of Action, and Recommended Daily Intake

### 3.1. Vitamin D Chemistry and Metabolism

The discovery of vitamin D dates back to the beginning of the nineteenth century and stems from the observation made by Sir Edward Mellanby about the significantly high incidence of rickets in the United Kingdom, especially in Scotland at that time [6]. McCollum identified rickets as a dietary deficiency disease and after some research, called “vitamin D” the factor capable of curing rickets [7]. The identification of vitamin D structure was achieved in the following years. Askew and colleagues isolated vitamin D2 in 1932, Windaus and Bock identified 7-dehydrocholesterol (7-DHC) in 1935, and finally, Windaus and Bock described the structure of vitamin D3 in 1937 [6].

Vitamin D comes in two forms. The first form, vitamin D3 (cholecalciferol), is synthesized from 7-DHC in the skin when exposed to ultraviolet (UV) light. When 7-DHC is irradiated, it is converted to previtamin D3; then skin temperature generates a thermal reaction and previtamin D3 is converted to vitamin D3. This last process permits the skin to continuously synthesize D3 and release it in the blood for up to 3 days after a single exposure to sunlight [8]. The second form, vitamin D2 (ergocalciferol) is derived from the fungal sterol ergosterol through UV-B irradiation. D2 differs from D3 in a double bond between C22 and C23 and a methyl group in the side chain at C24. The two forms are generally regarded as equivalent, but the difference in the side chains, and consequently in the hydroxylation sites, leads to a direct metabolization of vitamin D2 to 24(OH)D, which results in a lower affinity for hepatic 25-hydroxylase, vitamin D binding protein (DBP), and vitamin D receptor (VDR) [9].

Vitamin D is converted to the active form by Cytochrome (CYP) enzymes through 25-hydroxylation in the liver and 1α-hydroxylation mainly in the kidney. First, vitamin D is metabolized to 25(OH)D by a set of 25-hydroxylases: CYP27A1, the only mitochondrial enzyme characterized by vitamin D 25-hydroxylase activity, and the microsomal CYP2R1, CYP2J2/3, CYP3A4, CYP2D25, and CYP2C11 [10,11]. Interestingly, the microsomal enzyme CYP2R1 is known to be able to 25-hydroxylate not only D3 but also D2, whereas CYP27A1 can only 25-hydroxylate D3 [12]. Also, CYP2R1 exhibits a 7-fold higher affinity toward vitamin D3 and 26-fold higher overall activity than CYP27A1 [11]. After 25-hydroxylation, 25(OH)D is converted to 1,25-dihydroxyvitamin D [1,25(OH)_2_D], the active metabolite that circulates bound to plasma DBP and exerts its endocrine actions. CYP27B1 represents the only enzyme that has this 1α-hydroxylase activity. This enzyme is located mainly in the kidney, but also in other tissues, such as skin, brain, lungs, eyes, and breasts, even though the regulations of the extrarenal and renal CYP27B1 activities are different [13]. The regulation of the renal CYP27B1 is very tight: parathyroid hormone (PTH) stimulates 1α-hydroxylase, while fibroblast growth factor 23 (FGF23) and 1,25(OH)_2_D itself inhibit CYP27B1. High levels of calcium suppress CYP27B1 through suppression of PTH, and similarly, high phosphate levels suppress CYP27B1 by stimulating FGF23. Indirectly, 1,25(OH)_2_D increases FGF23 production and reduces 1,25(OH)_2_D levels inducing the catalytic enzyme CYP24A1, resulting in the inhibition of PTH and the consequent limitation of CYP27B1 activity [14,15]. 

1,25(OH)_2_D levels are regulated through a co-work of CYP27B1 and CYP24A1. CYP24A1 can hydroxylate also 25OHD, even though 1,25(OH)_2_D is the preferred substrate, and it is expressed in many target cells containing the VDR, suggesting its negative feedback role on the 1,25(OH)_2_D [16]. Interestingly, CYP24A1 has both 24 and 23-hydroxylase activity: the first produces the biologically inactive calcitroic acid, while the latter results in the biologically active 1,25(OH)_2_D_3_-26,23-lactone [17,18]. 

Furthermore, it should be mentioned that in the plasma membrane of the keratinocyte previtamin D_3_ can undergo a UVB-mediated isomerization to tachysterol_3_ (T_3_) and lumisterol_3_ (L_3_) [19].

An alternative pathway for vitamin D activation has been recently associated with CYP11A1, the side-chain cleavage enzyme essential for steroidogenesis. CYP11A1 20-hydroxylates vitamin D to the metabolite 20,23(OH)_2_D, which seems to have a similar activity to 1,25(OH)_2_D, for several functions [20]. In fact, 7-DHC, ergosterol, L_3_, and vitamins D_3_ and D_2_ also serve as substrates for CYP11A1 [21]. and this leads to the production of several metabolites, including 20(OH)D_3_, 22(OH)D_3_, 20,23(OH)_2_D_3_, 20,22(OH)_2_D_3_, 1,20(OH)_2_D^3^, 1,20,23(OH)_3_D_3_, 17,20,23(OH)_3_D_3_ [22]. 20(OH)D_2_, 17,20(OH)_2_D_2_ and 1,20(OH)_2_D_2_ [21]. Both CYP11A1 and CYP27B1 are involved in this alternative pathway that seems particularly relevant in placenta and adrenal glands which are characterised by high CYP11A1 expression [22].

Serum concentrations of 20(OH)D_3_ and 22(OH)D_3_ have been demonstrated to be only 30- and 15-fold lower than 25(OH)D_3_, respectively. On the basis of their concentrations and biological activity, their “in vivo” hormonal activity has been suggested [23]. Interestingly, alternative nuclear receptors for CYP11A1-derived D_3_-hydroxyderivatives have been identified [24], which include retinoid-related orphan receptors (ROR)α and γ [25], the arylhydrocarbon receptor (AhR) [26] and liver X receptors (LXRs) α and β [27] CYP11A1 and CYP27A1 also hydroxylate T_3_, leading to the production of 20S(OH)T_3_ and 25(OH)T_3_, respectively. T_3_ and 25(OH)T_3_ activate VDR, whereas 20S(OH)T_3_, and to a much lesser extent 25(OH)T_3_ and T3, activate AhR, LXR α and β, and the peroxisome proliferator-activated receptor γ (PPARγ) [19]. The skeleton serves as a sensor of the phosphate levels: to control hyperphosphatemia, osteocytes and osteoblasts respond to 1,25(OH)_2_D -VDR expressing and releasing FGF23 and repressing CYP27B1, while they induce CYP24A1 for feedback lowering 1,25(OH)_2_D levels [28]. Even if the primary effect of 1,25(OH)_2_D-VDR is to promote bone resorption [29], exploiting the catabolic action of vitamin D, it exerts also an anabolic action. 1,25(OH)_2_D induces osteopontin (SPP1 gene), increases osteoblast survival, and triggers ossification stimulating osteoblast proliferation [30]. Moreover, 1,25(OH)_2_D targets the osteocalcin (bone Gla protein, BGP) in osteoblasts, which is important for bone robustness and fracture resistance [31]. In the small intestine, 1,25(OH)_2_D-VDR action stimulates calcium absorption with both active and passive mechanisms [32]. These processes depend mainly on the VDR and CYP27B1. In particular, in the kidney CYP27B1 is induced by PTH under low-calcium conditions, and is repressed in a short feedback loop by 1,25(OH)_2_D, and in a long feedback loop by FGF23. Moreover, calcium is reabsorbed actively in the distal renal tubules, through a process similar to 1,25(OH)_2_D-VDR-induced duodenal calcium absorption [33].

### 3.2. Vitamin D Recommended Daily Intake

The prevalence of vitamin D deficiency in the general population is usually assessed by referring to the data of the US National Institutes of Health-led Vitamin D Standardization Program (VDSP) which sets the serum level of 25(OH) D < 30 nmol/L as the reference for deficiency [34]. Vitamin D deficiency seems particularly concerning in the European population, reaching 13% [35], while lower prevalence is found in the North American populations [36,37]. Moreover, the prevalence of vitamin D deficiency in the elderly seems significantly higher compared to childhood [37,38] and in dark-skinned ethnic groups compared to their white counterparts [35,39].

When vitamin D recommended requirements guidelines are considered, partially conflicting opinions can be found in the literature [40,41,42,43,44,45]. A further complication arises from the confusion existing between nutritional vitamin D guidelines targeted for the general population from clinical vitamin D guidelines intended for patient care [46].

Currently, available recommendations on daily intake of vitamin D are mainly based on musculoskeletal outcomes. An extensive comparison of vitamin D recommended daily intakes by the different national health authorities goes beyond the scope of this review and can be found somewhere else [46]. From a broad perspective, we can acknowledge that a vitamin D intake of 400 to 800 IU per day is recommended by the health authorities. However, it has been highlighted that these vitamin D intakes are not generally taken by the general population, therefore systematic vitamin D food fortification is performed by some countries [47,48,49,50,51], such as the United States, Canada, and Finland. Finally, it should be highlighted that vitamin D supplementation is recommended in some countries with doses and periods of supplementation partially differing among them [40].

## 4. The Role of Vitamin D: Gene Transcription and Immune Response

Nutrigenomics represents the subject that studies how the quality of diet, food intake, and lifestyle, influence the expression of the genome [52]. At a genomic level, all the 1,25(OH)_2_D actions are mediated by the VDR. The VDR is made up of three domains, the N-terminal DNA binding domain with two zinc fingers that bind to the grooves of the DNA at discrete sites named vitamin D response elements (VDREs), the C-terminal ligand binding domain, and the hinge region connecting these two domains together. VDR binds to its VDRE, causing the recruitment of coregulatory complexes, that can be both gene and cell-specific. VDR binding sites can be anywhere in the genome, often many thousands of base pairs away from the gene being regulated [53]. VDR regulates also nongenomic activities in bone cells, intestinal enterocytes, kidneys, colonocytes, and skin.

1,25(OH)_2_D directly influences the epigenome and transcriptome at thousands of loci within the human genome [54]. The ability of Vitamin D to modify gene transcription was initially highlighted with the discovery of VDR in the intestine [55]. and other tissues, including parathyroid glands, kidney, and bone [56], and by its similarity with other steroid hormone receptors [57].

VDR forms heterodimers with the retinoid X receptor alpha (RXR) to control the transcription of target genes [58], and this is important to achieve an adequate DNA-binding affinity [59,60]. There are many situations in which VDR acts independently of RXR and it uses other nuclear proteins as alternative cooperative binding partners on genomic DNA [61,62], or indirectly binds to DNA through other transcription factors [63].

VDR binding sites can be found within cis-regulatory modules (CRMs) which often contain sites for multiple transcription factors whose functional activities can be synergistic [64]. VDR interacts with a multiprotein complex containing co-receptors (i.e., RXR), pioneer factors (i.e., PU.1, CEBPα, GABPα, ETS1, RUNX2, BACH2), chromatin modifiers (i.e., KDM1A, KDM6B), chromatin remodelers (i.e., BRD7, BRD9), co-activators (i.e., MED1) and co-repressors (i.e., NCOR1, COPS2) [59].

1,25(OH)_2_D may also have suppressive activities in some circumstances, as evidenced by the suppressive effects on the IL-17A gene [65], where 1,25(OH)_2_D-VDR-RXR complex inhibits IL-17A production with multiple mechanisms: competition with NFAT binding to the IL-17A promoter [66], inhibition of Smad7 transcription [67], sequestration of Runx1 by VDR, and induction of Foxp3 (which associates with and inhibits NFAT and Runx1) [68].

1,25(OH)_2_D appears to have numerous epigenetic effects too [69]. Important genes involved in the vitamin D signalling system, including VDR, CYP2R1, CYP27B1, and CYP24A1 have large CpG islands in their promoter regions and this makes them sensitive to silencing by methylation [70]. Moreover, VDR interacts with coactivator and corepressor proteins, which in turn can influence chromatin modifiers, such as histone acetyltransferases (HATs), histone deacetylases (HDACs), histone methyltransferases (HMTs), and chromatin remodelers [70]. Furthermore, certain VDR ligands have DNA demethylating effects [70].

1,25(OH)_2_D can modify chromatin accessibility [71] through a direct ligand-dependent interaction of VDR [72] with chromatin-modifying enzymes [62], including HATs [73], HDACs [74], lysine demethylases such as KDM6B [75] and KDM1A [76] or chromatin remodelling proteins such as bromodomain-containing protein 7 (BRD7) and BRD9 [77].

**Figure 1:**
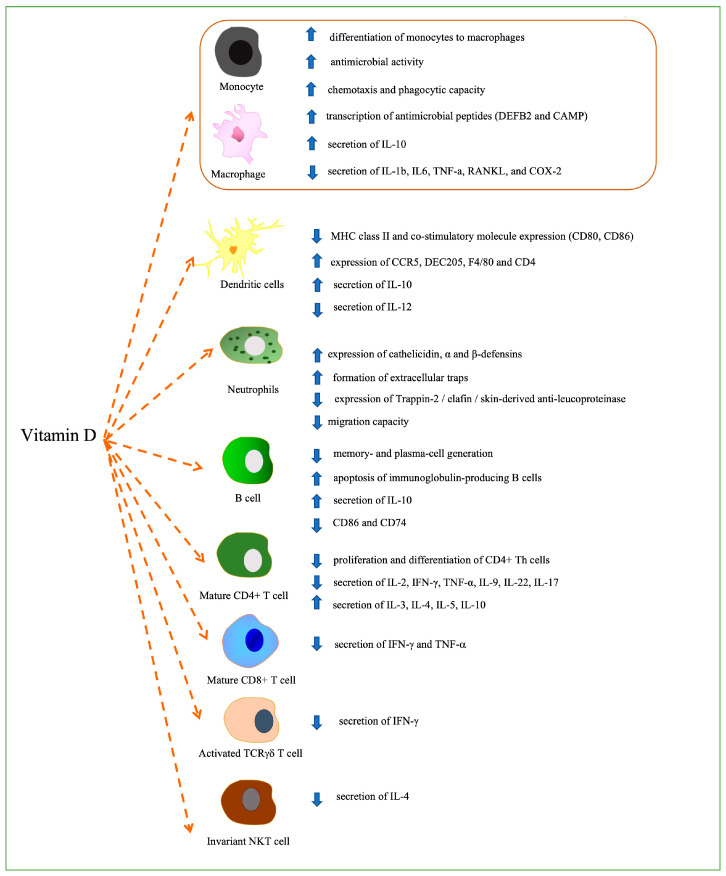
The effects of vitamin D on immune cells.

The recently highlighted ability of vitamin D to modulate innate and adaptive immunity [78] seems very interesting from a possible therapeutic perspective in several pathologies [65]. Immune cells can convert 25(OH)D into 1,25(OH)_2_D [79,80,81,82,83] and upregulate the CYP27B1 enzyme [84]. The effects of 1,25(OH)_2_D on immune cells depend on the state of cellular activation, as demonstrated in T cells, where VDR expression reaches its maximum 48 hours after activation [85] or in monocytes with a decrease of VDR expression after differentiation into macrophages and dendritic cells. The effects of vitamin D on immune cells are summarized in Figure 1.

Firstly, the differentiation of monocytes to macrophages has been shown to be promoted by vitamin D [86]. Vitamin D has also been demonstrated to increase the antimicrobial activity of these cells against pathogens, including Mycobacterium tuberculosis as well as their chemotaxis and phagocytic capacity [87]. In these cells, the 1,25(OH)_2_D-VDR-RXR complex seems to directly activate the transcription of antimicrobial peptides such as defensin β2 (*DEFB2*) and cathelicidin antimicrobial peptide (*CAMP*) [88,89,90]. Vitamin D induces the secretion of IL-10 and reduces the secretion of many pro-inflammatory factors, including IL-1b, IL-6, TNF-α, RANKL, and COX-2 [91] in macrophages. These inflammatory cytokines are downregulated through the upregulation of mitogen-activated protein kinase (MAPK) phosphatase(MKP)-1 and through the subsequent inhibition of LPS-induced p38 activation [92] or through inhibition of COX-2 expression by targeting thioesterase superfamily member 4 [93].

Vitamin D exerts an important effect also on dendritic cells (DCs). A more tolerogenic-immature DC phenotype characterised by decreased levels of MHC class II and co-stimulatory molecule expression (CD80, CD86) and increased expression of CCR5 (chemokine receptor), DEC205 (antigen-uptake receptor), F4/80 (macrophage marker), and CD40 [94] is induced by vitamin D, that can also suppress IL-12 secretion and increase the production of IL-10 [95,96,97,98,99].

Vitamin D also increases the antimicrobial activities of neutrophils by upregulating the expression of cathelicidin, α, and β-defensins [100] and inducing the formation of extracellular traps (NETs, trap and kill pathogens) [101]. Moreover in neutrophils vitamin D has been documented to reduce the expression of Trappin-2/elafin/skin-derived anti-leucoproteinase [102] as well as a reduced migration capacity [102].

The effects of vitamin D are also important for T and B cells. In B cells vitamin D inhibits memory and plasma cell generation and promotes the apoptosis of immunoglobulin-producing B cells [103,104]. Furthermore, 1,25(OH)_2_D can directly bind the IL-10 promoter region and upregulate IL-10 production and downregulate CD86 and CD74, suggesting in the first case a lower stimulation of the T cells [105] and in the second case a modulation of MHC-II [106].

Naïve T cells do not respond to vitamin D and the expression of VDR is induced by TCR signaling [107]. In mature T cells 1,25(OH)_2_D can have suppressive effects on the proliferation and differentiation of CD4+ T helper cells [108]. 1,25(OH)_2_D also inhibits the secretion of proinflammatory Th1 (IL-2, interferon-γ, tumor necrosis factor α), Th9 (IL-9), Th22 (IL-22) [109,110,111,112,113,114] and Th17 (IL-17) [115] cytokines while promoting the production of Th2 cytokines (IL-3, IL-4, IL-5, IL-10) [116].

Similar data are reported for CD8+ T cells, with downregulation of the production of IFN-γ and TNF-α [117].

Whether 1,25(OH)_2_D induces Tregs differentiation in humans is a matter of debate [118,119]. 1,25(OH)_2_D inhibits IFN-γ production by activated TCRγδ, T cell [120] and stimulates the production of IL-4 from Invariant NKT cells [121].

## 5. Vitamin D and Neurological Diseases: Current Evidence

This section discusses the available evidence in relation to the role of vitamin D deficiency in the pathogenesis of the main neurological diseases. Therapeutic implications are also discussed, when available. According to recent opinions, vitamin D status should not be considered as the level of serum 25(OH)D alone, but as a lifestyle marker in general. In fact, low vitamin D levels may reflect dietary habits, levels of physical exercise and can be influenced by many health factors including body mass index. Therefore, serum 25(OH)D levels should not be considered only as an indicator of vitamin D status, but also as a marker of good health [122]. However, when discussing current evidence, it is clear that most trials do not clarify whether lifestyle indicators were taken into consideration, often making it difficult to interpret the results from the point of view of etiological clarification [122].

### 5.1. Alzheimer’s Disease

AD represents the most prevalent type of neurodegenerative dementia. It usually starts with memory impairment and can progress to severely compromise the ability to carry out daily activities [123]. From a pathological perspective, AD is characterized by extracellular amyloid beta (Aβ) deposition, pathologic intracellular tau protein tangles, and neuronal loss [124].

Historically, many factors [125,126,127,128,129,130,131,132,133,134,135,136] have been called upon to explain the process of neurodegeneration associated with AD including the amyloid hypothesis, tau propagation hypothesis, mitochondrial dysfunction, and inflammation. Accumulating evidence is highlighting the role of vitamin D deficiency in AD. The rationale for the role of vitamin D in normal cognition is based on the specific functions of this vitamin. 1,25(OH)_2_D enhances the amyloid plaque’s phagocytosis and clearance by the immune cells, especially macrophages [137,138,139]. Moreover, transforming growth factor-beta-1 (TGF-beta), which represents a key regulator of the amyloid precursor protein (APP) expression (the APP promoter binding beta site is responsive toTGF-beta) [140], causes activation of SMAD proteins acting as coactivators or transcription factors in the nucleus [141]. Smad3, one of the SMAD proteins downstream in the TGF-beta signalling pathway, is known to be a specific coactivator for ligand-induced VDR transactivation, by forming a complex with a member of the steroid receptor coactivator-1 protein family in the nucleus [141]. Thus, Smad3 may mediate cross-talk between vitamin D and TGF-beta signalling pathways.

Finally, vitamin D has an important role in modulating the inflammatory response, intracellular oxidative stresses, and mitochondrial respiratory function [142]. These effects may be important in AD pathogenesis [143,144].

Several studies aimed to explore the association between vitamin D deficiency and the occurrence of AD, but they are burdened with many drawbacks, including the differences among the methods used for Vitamin D assessment, the high variability of the cut-offs used to define Vitamin D deficiency and insufficiency, and the diagnostic criteria used for cognitive impairment and dementia. With this in mind, the majority of the studies found that subjects with low 25(OH)D serum levels have a higher risk of developing AD [145,146,147,148,149,150,151,152,153], with genetically increased 25(OH)D levels found associated with reduced AD risk in individuals aged 60 years and over [154], albeit this still represents a matter of discussion, as it has not been confirmed by other studies [155,156] and some meta-analyses [157,158,159].

There is less uncertainty when the effects of vitamin D supplementation in AD patients are considered, with the vast majority of the studies finding a lack of benefit [160,161,162,163,164,165,166,167,168] and only a few of them showing promising results [169,170]. There is also evidence on a worsening effect of Vitamin D supplementation on AD progression [171]. Nonetheless, vitamin D supplementation represents a therapeutic strategy that warrants further investigation in AD patients.

### 5.2. Parkinson’s Disease

With a worldwide incidence estimate ranging from 5 to more than 35 new cases per 100,000 individuals yearly, PD represents the second-most common neurodegenerative disorder [172]. Although PD is well characterized from a neuropathological point of view, with the specific coexistence of the loss of pigmented dopaminergic neurons in the substantia nigra and the deposition of α-synuclein in neurons, the pathophysiological mechanisms are still not completely clarified, involving intracellular homeostasis of α-synuclein, mitochondrial dysfunction and neuroinflammation [172]. The emerging role of vitamin D in the cellular mechanisms of proliferation, differentiation, and immunoregulation, has led to the hypothesis of its contribution to PD [173].

Regarding the association between 25(OH)D concentrations and the risk of developing Parkinson’s disease no definitive results are available and the literature presents controversial conclusions. The first study was conducted by Knekt and colleagues, based on the Mini–Finland Health survey, and carried out in 40 areas of Finland with the recruitment of 3173 participants representing Finnish adults aged 30 years and over. The subjects were free from PD and did not use antipsychotic medication at baseline and during a 29-year follow–up 50 PD cases were identified. In this cohort, low serum 25(OH)D concentrations predicted an elevated risk of incidence of this neurodegenerative disease [174]. Lately, two large studies failed to confirm the correlation between 25(OH)D concentrations and Parkinson’s disease risk. In particular, Shrestha and colleagues, using data from the Atherosclerosis Risk in Communities (ARIC) Study, did not identify this association assessing 12,762 participants [175]. Similarly, in the Parkinson’s Associated Risk Syndrome (PARS) study, Fullard et al. found no data to support the hypothesis that chronic vitamin D insufficiency contributes to the pathogenesis of PD [176].

A possible explanation for the conflicting conclusions in the aforementioned studies could be found in the geographical and behavioural differences of the investigated populations. A different way to investigate the association between vitamin D and PD risk is to study VDR polymorphisms. In this context, a comprehensive meta-analysis by Wang et al. demonstrated that SNP FokI is associated with a decreased risk of PD in Asian populations but not in Caucasian populations [177].

One of the most consistent findings in the literature is the association between serum 25(OH)D concentrations and motor symptom severity in PD, assessed by Unified Parkinson’s Disease Rating Scale (UPDRS) and the Hoehn and Yahr stage (H&Y). Compared to what was previously reported for PD incidence, there is more agreement in the literature regarding this association. Several authors documented an inverse association between 25(OH)D concentrations and motor symptom severity [178,179,180,181]. Although a possible bias could be the so-called “reverse causation” (i.e., lower sun exposure in patients with more severe motor symptoms) data in the literature documented the non-occurrence of a decrease in 25(OH)D concentrations during the progression of PD [182].

Few data are available about the association between vitamin D and non-motor symptoms in PD. Overall, the published research documented a worsening of these symptoms (sleepiness, olfactory dysfunction, cognitive decline) in association with low 25(OH)D [183,184,185]. In most cases, however, the published results would need to be confirmed by further studies.

Although falls may be attributed to a wide range of etiologies, it is interesting to explore the effectiveness of vitamin D supplementation in preventing falls.

Vitamin D influence muscle strength and vitamin D insufficiency has been associated with a worse physical performance, increased bone resorption, decreased bone mineral density and therefore with an increased risk of fracture [186]. However, the studies exploring the effectiveness of vitamin D supplementation in preventing falls have heterogeneous results and, as highlighted in a recent meta-analysis, further research is needed to determine the relationship between 25(OH)D concentration and falls [186]. A metanalysis conducted by Bischoff-Ferrari et al. showed that vitamin D reduced the risk of falls among healthy ambulatory or institutionalized older individuals by 22% [187]. A Cochrane review suggested that vitamin D did not appear to reduce falls [188]. On the contrary, a recent meta-analysis [186] showed that daily doses of 700 IU to 2000 IU of supplemental vitamin D are associated with a lower risk of falling among ambulatory and institutionalized older adults, but this benefit appears small and might depend on additional calcium supplementation.

In light of the above data, although sometimes inconclusive, vitamin D supplementation might have positive effects in PD patients by reducing neuronal damage and limiting the phenomena of neuroinflammation. The results of a randomized placebo-controlled clinical trial, reported by Suzuki and colleagues and aiming to evaluate the relationship between 1200 IU/day of vitamin D administration and PD disease progression for a two years follow-up, documented a better neurological outcome in patients who received vitamin D [189]. However, these results were not confirmed by another study [190]. In conclusion, although there is limited evidence in favour of vitamin D supplementation in PD, the potential beneficial effects and the limited associated risks still suggest considering it. It will be necessary, in the future, for this therapeutic option to be supported by more consistent clinical trial data.

### 5.3. Amyotrophic Lateral Sclerosis

ALS is a heterogeneous multisystem neurodegenerative disorder characterized by the progressive degeneration of both upper and lower motor neurons. With an estimated annual incidence and prevalence in Europe ranging respectively from 2 to 3 cases per 100,000 and 10 to 12 per 100,000 individuals, similarly to other neurodegenerative diseases, ALS presents a complex pathogenetic architecture with a combination of genetic causes, environmental and lifestyle factors, and aging-related dysfunction. The underlying pathogenetic mechanisms include impaired protein homeostasis, aberrant RNA metabolism, cytoskeletal disturbances, axonal transport defects, impaired DNA repair, excitotoxicity, oligodendrocyte degeneration, neuroinflammation, and mitochondrial dysfunction. There is currently no effective treatment available for this neurological disorder, with only two compounds (riluzole and edavarone) approved as disease-modifying drugs. The currently employed therapeutic options focus on the symptomatic management of disease manifestations with a multidisciplinary approach to care, comprising neurologists, pulmonologists, psychologists, nutritionists, physical therapists, and specialized nurses, to improve the patient’s quality of life [191,192].

Despite the growing number of published studies, the role of vitamin D in ALS patients is a controversial issue. The lack of definitive results regarding this aspect is due to the frequent low methodological quality that has distinguished the majority of the research carried out so far. Systematic reviews and meta-analyses can help clarify the relationship between vitamin D and this neurodegenerative disease. In particular, Lanznaster and colleagues analysed studies reporting data about the role of vitamin D level as a biomarker for ALS diagnosis, for vitamin D level as a prognostic factor, or regarding the vitamin D supplementation effect on clinical outcomes. The authors considered clinical trials, cohort studies, or case-control studies, including 13 research articles [193]. With regards to the prevalence of vitamin D deficiency in ALS patients, although overall these subjects presented lower 25(OH)D concentrations compared with the normal range, no articles reported a significant difference in 25(OH)D concentrations between ALS patients and controls [194,195,196]. Only Cortese et al. reported a significant difference in an abstract, but these data were not subsequently confirmed in a full article [197]. About the prognosis, the analysis of the overall data from the studies analysed by Lanznaster and collaborators [195,196,198,199] did not document a correlation between vitamin D deficiency and motor dysfunction assessed by ALS Functional Rate Score—Revised (ALSFRS-R). Focusing on survival data, the data are conflicting. Blasco et al. showed a deleterious prognosis effect of higher 25(OH)D concentrations, independently of BMI, suggesting a direct effect, independently from the nutritional status [199]. On the contrary, Camu et al. support a neuroprotective function of vitamin D on motoneurons, reporting a positive association between vitamin D status and life expectancy [200]. In a further article, published by Yang et al., 25(OH)D concentrations were not correlated to the survival of ALS patients [201]. More recently, Juntas-Morales and colleagues performed a prospective study in which vitamin D deficiency was an independent prognostic factor for subjects affected by ALS [202].

In relation to the association between VDR polymorphisms and ALS, it has been reported that ApaI A allele was more frequent in the ALS group than in the control group and may be an ALS risk factor [203]. Furthermore, the BB genotype of the intronic Bsml polymorphism between exons 8 and 9 may be related to the disease [204]. Calcium absorption and lead toxicity caused by an alteration of VDR function have been hypothesised [204].

Regarding the beneficial effects of vitamin D supplementation in ALS patients, there are currently insufficient data to demonstrate its efficacy [196,205,206].

### 5.4. Multiple Sclerosis

MS is an immune-mediated demyelinating disease of the central nervous system and the most common acquired disabling neurological disease affecting young adults in developed countries [207]. The majority of MS patients experience an initial relapsing-remitting course, characterized by episodes of neurological worsening, followed by full or sometimes partial remission. After a variable period, the disease may enter into a secondary progressive phase characterized by gradual worsening of disability [208]. A minority of MS patients, diagnosed with primary progressive MS, experience a progressive disease from the beginning and show a continuous irreversible increase of functional impairment over the years [209].

There is strong evidence supporting the role of vitamin D in the pathogenesis and in influencing the course of MS. The first clue regarding the possible role of vitamin D in the pathogenesis of MS came from the so-called “latitude gradient” [210]. In fact, several epidemiology studies demonstrated that MS frequency increases with increasing latitude [211,212,213,214,215]. Furthermore, the prevalence of MS is lower than expected in those populations living at high latitudes eating high amounts of vitamin D-rich fatty fish [216,217]. However, it should be noted that this “latitude gradient” is progressively disappearing in the last decades and this has been related to an increased trend toward reducing sun exposure even in warmer climates, widespread use of sunscreens, and reduced time spent outdoors [210]. Sun exposure seems to influence the MS risk also during childhood, as the risk of developing MS is higher in those individuals who spent a lower amount of time outdoors during childhood and adolescence in the summer [218].

25(OH)D concentrations have been demonstrated to be lower in patients who develop MS, before the onset of the disease and this should be viewed as a risk factor rather than a consequence of MS [219,220]. Several studies tried to define the links between vitamin D insufficiency and disease course in patients affected by MS. Some studies found 25(OH)D concentrations correlated with clinical disability [221,222,223,224,225,226] whereas others did not [227,228]. Moreover, it seems that a low vitamin D status at the time of MS diagnosis may be associated with early conversion to secondary progression [229].

Furthermore, there is a general agreement that 25(OH)D concentrations may correlate with disease activity [223,226,230,231,232], albeit the effects of vitamin D supplementation on the relative risk of relapse in MS are less clear [233]. There is evidence from Mendelian randomisation studies which have shown that 25(OH)D is inversely associated with risk of MS as well as relapse [234,235,236].

Whether 25(OH)D concentrations differ among the different forms of MS is a matter of debate with some authors reporting higher values in relapsing-remitting MS patients than in progressive forms of MS [221,223] and others showing no significant differences [222].

An interesting and well-performed systematic meta-analysis analysed the clinical trials assessing the efficacy of vitamin D supplementation in MS and clinically isolated syndrome (CIS)/optic neuritis. Unfortunately, the analysis is burdened by the enrolment of a small number of patients, and by the high variability of the doses of vitamin D supplementation utilized. The 12 trials identified [237,238,239,240,241,242,243,244,245,246,247,248] demonstrated a significant elevation of serum 25(OH)D in treated patients, with a dose-response effect and with no increased risk of adverse effects [249]. The analysis of these trials showed no significant beneficial effects for vitamin D in these patients, with a possibility of an increased risk of relapse in MS patients on high doses of vitamin D supplementation. The Authors conclude that even if vitamin D supplementation may have a therapeutic role in the treatment of MS, these findings do not lead to the recommendation of vitamin D supplementation in MS, but further placebo-controlled clinical trials are needed.

The results are somehow overlapping when the most recent trials performed after McLaughlin’s meta-analysis are considered. The EVIDIMS trial compared the effects of every other day high- (20,400 IU) vs low-dose (400 IU) cholecalciferol supplementation on clinical and imaging markers of disease activity in relapsing-remitting MS and CIS patients [250] The Authors recognized that the sample size of this trial was underpowered but there was no significant difference in terms of clinical and MRI metrics (including lesion development, enhancing lesions, and brain atrophy) between the two groups, after 18 months.

Another clinical trial has recruited from March 2012 through April 2019 at 16 neurology clinics in the United States. 172 patients were assigned to low-dose (600 IU/day) versus high-dose (5000 IU/day) regimen of vitamin D3 as an add-on therapy to glatiramer acetate (Copaxone). Both therapeutic regimens were generally well tolerated. No significant difference was shown in terms of the proportion of subjects that experienced a relapse nor in terms of annualised relapse rate [251].

Interestingly, CHOLINE [248] and SOLAR [247] randomized controlled trials showed positive effects of vitamin D supplementation on secondary endpoints. More precisely, vitamin D supplementation resulted in a lower number of enlarging or new T2 lesions, new T1 lesions, as well as lower hypointense T1 lesion volume and disability progression.

On these bases, we can conclude that there is a general agreement that testing serum levels of 25(OH)D in patients with MS is useful due to the higher prevalence of low levels in this population. From this perspective, given the low bone mineral density frequently documented even at very early stages [252] and considering the higher risk of falls and fractures of MS patients [253,254], the correction of the low serum 25(OH)D concentrations should be always performed when documented [255]. There is a current agreement that vitamin D deficiency should be prevented in MS patients, and that levels around 100 nmol/L or somewhat higher should be achieved [255].

Uncertainty on the best dosage to advise still exists, albeit vitamin D3 supplementation from 1000–2000 IU/day [255] to 10,400 IU/day [242] is considered well tolerated and may be prescribed. However, as already highlighted, the higher doses should be reserved for MS patients living in North Europe and North America, whereas lower doses of supplementation may be prescribed to Australian and South European patients [255].

Indeed, it appears that the beneficial UVB effect could also be independent from the actual production of 25(OH)D_3_, and, from this perspective, phototherapy, alternatively or in parallel with vitamin D supplementation, has been proposed as a hypothetical treatment for MS patients [256].

### 5.5. Migraine

Migraine is among the most prevalent neurological conditions in the general population, with a worldwide burden constantly increasing in recent years and a higher impact in the most developed countries [257]. Clinically, migraine is characterized by headaches usually accompanied by nausea, vomiting, osmophobia, photophobia, and phonophobia. Migraine is more prevalent in females [257] and causes significant functional impairment, reduced health-related quality of life [258,259,260], and psychiatric comorbidities [261]. There is a significant gap between migraineurs who need preventive treatment and those who are actually on it [262], and this may be due to several reasons including the reduced tolerability of some medicines, their adverse event profile, and the presence of comorbidity [263,264,265].

The association between low vitamin D serum levels and headache has been found in several observational, cross-sectional, and case-control studies [266,267,268,269,270,271,272,273,274,275,276,277]. Interestingly, this association is not limited to migraine but has been highlighted also in other types of headaches, including tension-type headache in both adult [266] and pediatric [277] populations.

A recent study [278] found that vitamin D deficiency was more frequent in patients affected by chronic migraine–medication overuse migraine compared to those with episodic migraine or tension-type headache. This was independent of the season of evaluation and by the patient lifestyle or headache treatment.

However, the sixth survey of the Tromsø Study (Tromsø6) on 11,614 subjects found no significant association between migraine and serum 25(OH)D concentrations, but the risk of non-migraine headache was negatively associated with vitamin D serum levels [279]. Similarly, another study failed to demonstrate any significant differences in the serum 25(OH)D concentrations in migraineurs compared to controls [280] 25(OH)D levels are lower in patients with migraine and tension-type headache, but a straightforward temporal association is yet to be determined (i.e., if vitamin D deficiency can lead to headache or if headache might lead to vitamin D deficiency). In fact, in the literature, there is a lack of longitudinal investigations [281,282].

Vitamin D supplementation has also been tested in clinical trials in migraine patients [283,284,285,286]. In these trials, a variable dosage of vitamin D was used and the follow-up period was variable. However, a significant reduction in the number of headache attacks and improved frequency of the episodes was generally reported [283,284,285,286], but the effects on the intensity of pain were less evident [286].

### 5.6. Diabetic Neuropathy

Diabetic neuropathy represents the main complication of diabetes [287,288,289] and its clinical manifestations can be distinguished by distribution and [290] course. Its prevalence varies according to the duration of the disease with approximately 50% of diabetic patients eventually developing neuropathy [288].

Diabetic neuropathy is burdened by significant morbidity and is associated with the occurrence of diabetic foot ulcers, with possible infections, including osteomyelitis, Charcot neuroarthropathy, and amputations [291].

The most common form of diabetic neuropathy is distal symmetrical polyneuropathy, which is characterized by slowly progressive distal hypoesthesia due to the degeneration of sensory axons, followed, in the most advanced stages by distal paresis due to the late involvement of motor axons [289]. The typical sensory involvement has been classically described as “stocking-glove” hypoesthesia. The impact of diabetic polyneuropathy on the disease course can be very disabling with a significant reduction of the quality of life of the affected patients [292].

Among others, vitamin D deficiency has been recognized as a risk factor for the development of diabetic neuropathy [293,294], after the evidence that hypovitaminosis D is linked to the occurrence and severity of sensory polyneuropathy in these patients [294,295,296,297]. A recent meta-analysis has confirmed that vitamin D deficiency is highly prevalent among diabetic patients with neuropathy [298].

The mechanisms by which vitamin D deficiency could predispose diabetic patients to the onset of neuropathy are manifold and still largely remain speculative. They include atherosclerosis, worsening of insulin resistance, increased inflammation, and oxidative injury to blood vessels eventually leading to nerve ischaemia [294,299,300]. Furthermore, vitamin D has been demonstrated to have multiple trophic effects on the peripheral nervous system, including myelination, axonal homogeneity of peripheral nerves, and neuronal-cell differentiation [301].

Vitamin D deficiency has been involved in the pathogenesis of small-fiber neuropathy, particularly affecting nociceptor fibers [302], but also parasympathetic fibers, especially in younger patients affected by type 2 diabetes [303].

In relation to the supplementation of vitamin D, the results of the completed clinical trials seem quite encouraging [298]. Supplementation has been found to be beneficial for neuropathic pain as well as for the other symptoms of neuropathy [304,305,306,307,308,309,310]. A very interesting recent study has demonstrated that treatment with cholecalciferol 40,000 IU/week leads to a clinically significant improvement as well as improvement of cutaneous microcirculation and to a decrease in serum levels of IL-6 and an increase of serum IL-10 after 24 weeks of vitamin D supplementation [311].

It should be kept in mind that vitamin D deficiency also favours bone loss by stimulating parathyroid hormone secretion, with an increased risk of fracture, and that vitamin D supplementation of at least 800 to 2000 IU of vitamin D per day is currently generally recommended in any case for diabetic patients to reach serum 25(OH)D levels of 75 nmol/L since these levels prevent bone mineralization defects [312].

Table 1 summarizes the ongoing clinical trials exploring the effect of vitamin D supplementation in neurological diseases. Table 2 summarizes the results of the main completed clinical trials exploring the effects of vitamin D supplementation in neurological diseases.

## 6. Conclusions and Future Directions

Our review shows that a role of vitamin D in neurological diseases is biologically plausible. The effects of vitamin D on the course of different neurological diseases are only partly understood, and current and future clinical trials may clarify its possible therapeutic applications and the best dose required for each condition. Future trials may also clarify the role of routine vitamin D supplementation in the general population to reduce the risk of developing neurological diseases.

## Figures and Tables

**Table 1:** Ongoing clinical trials exploring the effect of vitamin D supplementation in neurological diseases. AD Alzheimer’s disease; BMI body mass index; CDR Clinical Dementia Rating; CIS clinically isolated syndrome; EDSS Expanded Disability Status Scale; FA fatty acids; GMFCS Gross Motor Function Classification System; HbA1c haemoglobin A1c; IU international unit; PD Parkinson’s disease; RRMS relapsing-remitting multiple sclerosis; STN-DBS subthalamic nucleus-deep brain stimulation. To convert 25(OH)D from ng/mL to nmol/L the value is multiplied by 2.5.

Formulation	Indication(s)	Status	Phase	Notes	References
Cholecalciferol	RRMS and serum vitamin D level below 25 ng/mL; age ≥ 18 years old	Recruiting	N/A	low dose vitamin D3 supplementation (800 IU daily for 6 months) or high dose vitamin D3 supplementation (50,000 IU weekly dose)	NCT03610139
Calcifediol or Cholecalciferol	RRMS and Vitamin D deficiency/insufficiency [25(OH)D < 30 ng/mL]; age ≥ 18 years old	Recruiting	IV	calcifediol 50 micrograms per day or cholecalciferol 50 micrograms per day, for 24 weeks	NCT05340985
Vitamin D	AD; age 50 to 90; CDR 0.5 to 1	Recruiting	N/A	Behavioural interventions (therapeutic lifestyles changes) and diet (intake of micronutrient supplements consisting of multivitamin, vitamin D, calcium, and phosphorus)	NCT03860792
Cholecalciferol	CIS or RRMS; age 18 to 45; EDSS of 5.5 or less; patients must demonstrate features of a first typical optic neuritis within 21 days of recruitment	Recruiting	II	5 days of high-dose oral vitamin D3 (50,000 IU daily × 5), followed by 85 days of moderate dose oral vitamin D3 (10,000 IU daily × 85 days)	NCT03302585
Cholecalciferol	Patient has had a classic CIS within the past 90 days	Active, not recruiting	III	Patients will receive 100.000 IU of cholecalciferol every 14 days for a maximum of 24 months	NCT01817166
Cholecalciferol	PD in STN-DBS treatment	Enrolling by invitation	N/A	Juvit D3 dosage based on the BMI: for BMI under 25–4000 IU/day; for BMI 25 to 30–5000 IU/day; for BMI over 30–6000 IU/day.	NCT04768023
Cholecalciferol	Painful diabetic neuropathy, insulin dependent diabetes or insulin independent diabetes; age 25 to 80 years; HbA1c level must be ≥6.5%	Recruiting	N/A	single oral dose capsule 200,000 IU of Cholecalciferol	NCT05080530
Cholecalciferol	episodic cluster headache as well as cluster periods that are predictable and have a duration of 6 weeks or greater and approxi-mately one attack daily minimum OR chronic cluster headache with approximately one attack daily	Recruiting	III	Vitamin D + multi-vitamin for 3 weeks. At the end of 3 weeks they will complete an online or paper questionnaire and blood work will be done.	NCT04570475
Vitamin D	Episodic migraine without or without aura; age 20 to 65; baseline migraine days 4 to 15 days per month, blood level of vitamin D < 30 ng/mL at baseline	Recruiting	N/A	Omega-3 FA (first 4-week) plus Vitamin D (second 4-week)	NCT05449145
Cholecalciferol	Cerebral palsy with spastic or mixed tone; GMFCS Level I-III (i.e., ambulatory); age 13 to 17	Enrolling by invitation	N/A	3 g β-hydroxy-β-methylbutyrate + 1000 IU of Vitamin D3 per day for 12 weeks	NCT05384951
Vitamin D	Painful diabetic neuropathy (diagnosis based on validated Diabetic Neuropathy Symptoms and Diabetic Neuropathy Examination); low vitamin D status at baseline (<30 ng/mL)	Recruiting	III	Vitamin D 5000 IU Oral Tablet once daily for 8 weeks	NCT04689958
1,25(OH)_2_D	Friedreich’s Ataxia with confirmed genetic diagnosis	Active, not recruiting	IV	1,25(OH)_2_D 0.25 mcg/24 h for a year, to evaluate the effects on the neurological symptoms.	NCT04801303

**Table 2:** Completed clinical trials exploring the effect of vitamin D supplementation in neurological diseases. ALS amyotrophic lateral sclerosis; ARR annualized relapse rate; CIS Clinically isolated syndrome; EDSS Expanded Disability Status Scale; IFN Interferon; IL interleukin; IU International Units; MS multiple sclerosis; ON optic neuritis; QoL quality of life; RNFL retinal nerve fiber layer; RRMS Relapsing Remitting Multiple Sclerosis; SC subcutaneous. To convert 25(OH)D from ng/mL to nmol/L the value is multiplied by 2.5.

Formulation	Indication(s)	Phase	Main Results	References
Cholecalciferol	2-year study, 181 RRMS patients with (1) a low serum 25-hydroxy vitamin D concentration (<75 nmol/L), (2) treatment with interferon beta-1a 44 μg (SC 3 times per week) 4 months ± 2 months before randomization, and (3) at least one documented relapse during the previous 2 years, randomized to oral cholecalciferol 100,000 IU or placebo every other week for 96 weeks	II	No change in the ARR at 96 week; good efficacy on MRI parameters (less new hypointense T1-weighted lesions; a lower volume of hypointense T1-weighted lesions, and a lower progression of EDSS)	NCT01198132 Cholecalciferol in relapsing-remitting MS: A randomized clinical trial (CHOLINE) [248].
Cholecalciferol	229 RRMS patients treated with SC IFN-β-1a 44 μg 3 times weekly and serum vitamin D levels <150 nmol/were included and randomized 1:1 to receive SC IFN-β-1a plus placebo (n = 116) or SC IFN-β-1a plus oral high-dose vitamin D3 14,007 IU/d (n = 113).	II	No significant difference in the proportion of patients with no evidence of disease activity at week 48. Better MRI outcomes (combined unique active lesions and change from baseline in total volume of T2 lesions) in those patients receiving SC IFN-β-1a plus oral high-dose vitamin D3	NCT01285401 Randomized trial of daily high-dose vitamin D3 in patients with RRMS receiving subcutaneous interferon β-1a [247].
Cholecalciferol	CIS patients and healthy control participants were randomised to: placebo, 5000 IU or 10,000 IU vitamin D3/day (Vigantol oil)	I/II	No immunological, MRI or clinical evidence of benefit over 24 weeks	NCT01728922 Effects of vitamin D3 in clinically isolated syndrome and healthy control participants: A double-blind randomised controlled trial [246].
Cholecalciferol	52 patients with confirmed unilateral ON aged 15–38 years and low serum vitamin D levels. Patients were randomly allocated to receive 6 months of treatment with adding either 50,000 IU/week vitamin D or placebo	II	In the 27 patients treated with vitamin D, no significant effect on the thickness of RNFL or macula was found.	NCT01465893 Effects of vitamin D on retinal nerve fiber layer in vitamin D deficient patients with optic neuritis: Preliminary findings of a randomized, placebo-controlled trial [245].
Ergocalciferol	23 adults with clinically active RRMS were randomized to 6 months’ double-blind placebo-controlled high-dose vitamin D2, 6000 IU capsules, dose adjusted empirically aiming for a serum vitamin D 130–175 nmol/L. All received daily low-dose (1000 IU) D2 to prevent deficiency	I/II	No significant therapeutic advantage in RRMS for high-dose D2 over low-dose D2 supplementation was found	ACTRN12606000359538 A randomized trial of high-dose vitamin D2 in relapsing-remitting multiple sclerosis [244].
Cholecalciferol	45 IFNβ-treated MS patients were recruited. 21 patients were assigned to 800 IU of vitamin D3 per day, while 24 patients received 4370 IU per day for one year.	IV	No significant change in flu-like symptoms. IL-17 levels were significantly increased in the low dose group, while patients receiving high dose vitamin D had a heterogeneous IL-17 response. No significant differences in relapse rate, EDSS, QoL, serum IL10 and IFNγ were found	NCT01005095 Vitamin D supplementation for patients with multiple sclerosis treated with interferon-beta: a randomized controlled trial assessing the effect on flu-like symptoms and immunomodulatory properties [243].
Cholecalciferol	40 patients with RRMS were randomized to receive 10,400 IU or 800 IU cholecalciferol daily for 6 months	I	Cholecalciferol with 10,400 IU daily is associated with reduction of IL-17 production by CD4^+^ T cells and decreased proportion of effector memory CD4^+^ T cells with concomitant increase in central memory CD4^+^ T cells and naive CD4^+^ T cells	NCT01024777 Safety and immunologic effects of high- vs. low-dose cholecalciferol in multiple sclerosis [242].
Cholecalciferol	35 adult and fully ambulatory RRMS patients were included in the vitamin D3 Supplementation with 20,000 IU weekly group and 33 in the placebo group	III	Supplementation with 20,000 IU vitamin D(3) weekly did not result in beneficial effects on the measured MS-related outcomes	NCT00785473 Effect of vitamin D3 supplementation on relapses, disease progression, and measures of function in persons with multiple sclerosis: exploratory outcomes from a double-blind randomised controlled trial [241].
Vitamin D	Peroral 20,000 IU once weekly as an add on therapy to IFNβ-1b vs placebo in patients with MS	IV	Vitamin D3 add on treatment to IFNB reduces MRI disease activity in MS (fewer new T2 lesions, T1 enhancing lesions). There was a tendency to reduced disability accumulation and to improved tandem gait. No significant differences in the ARR	NCT01339676 A randomised, double blind, placebo controlled trial with vitamin D3 as an add on treatment to interferon β-1b in patients with multiple sclerosis [240].
Cholecalciferol	50 patients with RRMS aged 25 to 57 years and normal serum 25-hydroxyvitamin D were randomly allocated to receive 12 months of treatment with either escalating 1,25(OH)_2_D doses up to 0.5 μg/day or placebo combined with disease-modifying therapy	II	Adding low-dose vitamin D to routine disease-modifying therapy had no significant effect on the EDSS score or relapse rate	N/A Effects of Adjunct Low-Dose Vitamin D on Relapsing-Remitting Multiple Sclerosis Progression: Preliminary Findings of a Randomized Placebo-Controlled Trial [239].
Alfacalcidol	Alfacalcidol (1 mcg/d, n = 80) or placebo (n = 78) was administered for six consecutive months in MS patients	N/A	Alfacalcidol decreased the fatigue score and improved QoLas compared with placebo. The Alfacalcidol-treated group had reduced number of relapses and higher proportion of relapse-free patients	(The trial was not registered as Alfacalcidol is considered a natural supplement and not a drug in Israel) Effect of Alfa-calcidol on multiple sclerosis-related fatigue: A randomized, double-blind placebo-controlled study [238].
Cholecalciferol	30 ON patients (15 in each of 2 groups, aged 20–40 years) with serum 25 hydroxyvitamin D levels of less than 30 ng/mL were enrolled. The treatment group received 50,000 IU of vitamin D3 weekly for 12 months and the control group received a placebo weekly for 12 months	N/A	Risk reduction of 68.4% for the conversion to MS after 12 months. Patients in the treatment group had a significantly lower incidence rate of cortical, juxtacortical, corpus callosal, new T2, new gadolinium-enhancing lesions and black holes. The mean number of total plaques showed a marginally significant decrease in the group receiving vitamin D3 supplementation as compared with the placebo group.	IRCT201205319919N1 Preventive effect of vitamin D3 supplementation on conversion of optic neuritis to clinically definite multiple sclerosis: a double blind, randomized, placebo-controlled pilot clinical trial [237].
Cholecalciferol	172 patients with RRMS, age 18 to 50 years, and EDSS ≤ 4.0, after completing a one-month run-in of glatiramer acetate, were randomized 1:1 to oral vitamin D3 5000 IU versus 600 IU daily	III	No significant difference was shown in terms of the proportion of subjects that experienced a relapse nor in terms of annualised relapse rate	NCT01490502 The vitamin D to ameliorate multiple sclerosis (VIDAMS) trial: study design for a multicenter, randomized, double-blind controlled trial of vitamin D in multiple sclerosis [251].
Cholecalciferol	The EVIDIMS trial compared the effects of every other day high- (20,400 IU) vs low-dose (400 IU) cholecalciferol supplementation on clinical and imaging markers of disease activity in 53 MS patients of which 41 completed the study.	II	The Authors recognized that the sample size of this trial was underpowered but no significant difference in terms of clinical and MRI metrics (including lesion development, enhancing lesions, and brain atrophy) between the two groups, after 18 months.	NCT01440062 High-dose vitamin D supplementation in multiple sclerosis—results from the randomized EVIDIMS (efficacy of vitamin D supplementation in multiple sclerosis) trial [250].
Cholecalciferol	4143 women aged 65 and older without probable dementia at baseline who participated in the WHI Calcium and Vitamin D Trial and the WHI Memory Study. 2034 women were randomized to receive 1000 mg of calcium carbonate combined with 400 IU of vitamin D3 (treatment) and 2109 to placebo.	III	no association between treatment assignment and incident cognitive impairment	Part of NCT00000611 Calcium and Vitamin D Supplementation and Cognitive Impairment in the Women’s Health Initiative [313].
Vitamin D supplements	43 white outpatients with a new diagnosis of AD, who had not taken anti-dementia drugs or vitamin D supplements were prescribed memantine alone (n = 18), vitamin D alone (n = 17), or memantine plus vitamin D (n = 8) for an average of 6 months. Vitamin D supplements were given orally, either daily or monthly. The dose ranged between 400 and 1000 IU per day, or 100,000 and 200,000 IU per month.	N/A	Patients with AD who were treated for 6 months with the combination of memantine plus vitamin D supplements had a statistically and clinically significant gain in global cognitive performance	N/A Effectiveness of the Combination of Memantine Plus Vitamin D on Cognition in Patients with Alzheimer Disease [314].
Cholecalciferol	5110 participants were randomized to receive vitamin D3 (n = 2558) or placebo (n = 2552). Oral vitamin D3 in an initial dose of 200 000 IU, followed a month later by monthly doses of 100 000 IU, or placebo for a median of 3.3 years (range, 2.5–4.2 years).	III	Negative results on stroke prevention	ACTRN12611000402943 Effect of Monthly High-Dose Vitamin D Supplementation on Cardiovascular Disease in the Vitamin D Assessment Study: A Randomized Clinical Trial [315].
Cholecalciferol	The experimental group received an add-on oral vitamin D 5000 IU once daily and standard treatment (pregabalin, gabapentin, or amitriptyline) over eight weeks. The control group received standard treatment alone.	II/III	The addition of oral vitamin D 5000 IU to standard treatment significantly improves pain, mood, and vitamin D levels more effectively than standard treatment alone in patients with diabetic neuropathy.	NCT04689958 The Benefits of Add-on Therapy of Vitamin D 5000IU to the Vitamin D Levels and Symptoms in Diabetic Neuropathy Patients: A Randomized Clinical Trial [304].
Cholecalciferol	A single intramuscular dose of 600,000 IU of vitamin D to 143 participants with predominantly type 2 diabetes	N/A	Treatment with a single intramuscular dose of 600,000 IU of vitamin D in patients with painful diabetic neuropathy is associated with a significant decrease in the symptoms of painful diabetic neuropathy	NCT02737423 Vitamin D for the treatment of painful diabetic neuropathy [207].
Cholecalciferol	112 type 2 diabetic patients with diabetic peripheral neuropathy and vitamin D deficiency were assigned to a treatment group (n = 57) and a placebo group (n = 55). Patients received either oral vitamin D3 capsules or starch capsules once weekly for 8 weeks	N/A	Short-term oral vitamin D3 supplementation improved vitamin D status and the symptoms of neuropathy in patients with type 2 diabetes.	N/A Prospective Evaluation of the Effect of Short-Term Oral Vitamin D Supplementation on Peripheral Neuropathy in Type 2 Diabetes Mellitus [306].
Cholecalciferol	60 type 2 diabetic patients with painful diabetic neuropathy were enrolled and received weekly 50,000 IU of vitamin D3 for 12 weeks orally	N/A	Oral supplementation of vitamin D 3 (50,000 IU) once weekly for 12 weeks was associated with improvement in the serum level of vitamin D and significant decrease in the symptoms and sign of diabetic neuropathy.	IRCT2017102325266N2 Dose vitamin D supplementations improve peripheral diabetic neuropathy? A before-after clinical trial [305].
Cholecalciferol	Patients with migraine. 24 weeks of vitamin D3 (24 patients, 100 μg/day) or placebo (24 patients)	III	Vitamin D3 was superior to placebo in reducing migraine days in migraine patients	NCT01695460 A randomized, double-blinded, placebo-controlled, parallel trial of vitamin D3 supplementation in adult patients with migraine [286].
Cholecalciferol	80 episodic migraineurs who randomly assigned into two equal groups to receive either daily dose of vitamin D3 2000 IU (50 μg) or placebo for 12 weeks	N/A	Improvement of headache characteristics and reduction of neuro-inflammation in episodic migraine	IRCT20151128025267N6 Vitamin D3 might improve headache characteristics and protect against inflammation in migraine: a randomized clinical trial [316].
Cholecalciferol	31 female and 26 male 5–15-year-old children with migraine headaches were randomly allocated to receive 2 mg/kg/day of topiramate or 2 mg/kg/day of topiramate plus one 500,000 IU vitamin D3 pearl weekly for two consecutive months	N/A	the combination of topiramate and vitamin D3 was more effective than topiramate alone in reducing the monthly headaches frequency and disability score	IRCT201701092639N20 Efficacy of topiramate alone and topiramate plus vitamin D3 in the prophylaxis of pediatric migraine: A randomized clinical trial [317].
Cholecalciferol	39 patients in intervention group and 38 patients in the control group were allocated with simple randomization method (Vitamin D 50,000 IU/week vs placebo) during 10 weeks	N/A	Mean headache frequency and headache diary result were lower among the intervention group compared to placebo group	IRCT2012122911763N4 Effect of Vitamin D supplementation on symptoms and C-reactive protein in migraine patients [284].
Cholecalciferol	57 adults with episodic migraine were randomized to simvastatin 20 mg tablets twice-daily plus vitamin D3 1000 international units capsules twice-daily or matching placebo tablets and capsules for 24 weeks	II	Efficacy of simvastatin plus vitamin D3 in decreasing the number of migraine days from the baseline period	NCT01225263 Simvastatin and vitamin D for migraine prevention: A randomized, controlled trial [285].
Cholecalciferol	48 ALS patients, 34 with deficient (<20 ng/mL) and 14 with insufficient (20–29 ng/mL) serum levels of vitamin D, were randomized and treated by 3 different doses of cholecalciferol [50,000, 75,000 and 100,000 IU /month] and evaluated after 6-months	N/A	no significant effects on motor dysfunction	Vitamin D supplementation has no effects on progression of motor dysfunction in amyotrophic lateral sclerosis (ALS) [205].

## Data Availability

Not applicable.

## References

[1] Ma Y., Khalifa B., Yee Y.K., Lu J., Memezawa A., Savkur R.S., Yamamoto Y., Chintalacharuvu S.R., Yamaoka K., Stayrook K.R. (2006). Identification and Characterization of Noncalcemic, Tissue-Selective, Nonsecosteroidal Vitamin D Receptor Modulators. J. Clin. Investig..

[2] Yeshokumar A.K., Saylor D., Kornberg M.D., Mowry E.M. (2015). Evidence for the Importance of Vitamin D Status in Neurologic Conditions. Curr. Treat. Opt. Neurol..

[3] di Somma C., Scarano E., Barrea L., Zhukouskaya V.V., Savastano S., Mele C., Scacchi M., Aimaretti G., Colao A., Marzullo P. (2017). Vitamin D and Neurological Diseases: An Endocrine View. Int. J. Mol. Sci..

[4] Koduah P., Paul F., Dörr J.M. (2017). Vitamin D in the Prevention, Prediction and Treatment of Neurodegenerative and Neuroinflammatory Diseases. EPMA J..

[5] Moretti R., Morelli M.E., Caruso P. (2018). Vitamin D in Neurological Diseases: A Rationale for a Pathogenic Impact. Int. J. Mol. Sci..

[6] DeLuca H.F. (2014). History of the Discovery of Vitamin D and Its Active Metabolites. BoneKEy Rep..

[7] McCollum E.V., Simmonds N., Becker J.E., Shipley P.G. (2002). The effect of additions of fluorine to the diet of the rat on the quality of the teeth. 1925. Studies on experimental rickets. XXI. An experimental demonstration of the existence of a vitamin which promotes calcium deposition. 1922. The effect of additions of fluorine to the diet of the rat on the quality of the teeth. 1925. J. Biol. Chem..

[8] Koshy K.T. (1982). Vitamin D: An Update. J. Pharm. Sci..

[9] Houghton L.A., Vieth R. (2006). The Case against Ergocalciferol (Vitamin D2) as a Vitamin Supplement. Am. J. Clin. Nutr..

[10] Sugimoto H., Shiro Y. (2012). Diversity and Substrate Specificity in the Structures of Steroidogenic Cytochrome P450 Enzymes. Biol. Pharm. Bull..

[11] Zhu J., Deluca H.F. (2012). Vitamin D 25-Hydroxylase—Four Decades of Searching, Are We There Yet?. Arch. Biochem. Biophys..

[12] Zhu J.G., Ochalek J.T., Kaufmann M., Jones G., DeLuca H.F. (2013). CYP2R1 Is a Major, but Not Exclusive, Contributor to 25-Hydroxyvitamin D Production in Vivo. Proc. Natl. Acad. Sci. USA.

[13] Adams J.S., Rafison B., Witzel S., Reyes R.E., Shieh A., Chun R., Zavala K., Hewison M., Liu P.T. (2014). Regulation of the Extrarenal CYP27B1-Hydroxylase. J. Steroid Biochem. Mol. Biol..

[14] Bikle D.D. (2014). Vitamin D Metabolism, Mechanism of Action, and Clinical Applications. Chem. Biol..

[15] Bikle D.D., Rasmussen H. (1975). The Ionic Control of 1,25-Dihydroxyvitamin D3 Production in Isolated Chick Renal Tubules. J. Clin. Investig..

[16] Jones G., Strugnell S.A., DeLuca H.F. (1998). Current Understanding of the Molecular Actions of Vitamin D. Physiol. Rev..

[17] Sakaki T., Sawada N., Komai K., Shiozawa S., Yamada S., Yamamoto K., Ohyama Y., Inouye K. (2000). Dual Metabolic Pathway of 25-Hydroxyvitamin D3 Catalyzed by Human CYP24. Eur. J. Biochem..

[18] Jones G., Prosser D.E., Kaufmann M. (2012). 25-Hydroxyvitamin D-24-Hydroxylase (CYP24A1): Its Important Role in the Degradation of Vitamin D. Arch. Biochem. Biophys..

[19] Slominski A.T., Kim T.K., Slominski R.M., Song Y., Janjetovic Z., Podgorska E., Reddy S.B., Song Y., Raman C., Tang E.K.Y. (2022). Metabolic activation of tachysterol3 to biologically active hydroxyderivatives that act on VDR, AhR, LXRs, and PPARγ receptors. FASEB J..

[20] Slominski A.T., Janjetovic Z., Fuller B.E., Zmijewski M.A., Tuckey R.C., Nguyen M.N., Sweatman T., Li W., Zjawiony J., Miller D. (2010). Products of Vitamin D3 or 7-Dehydrocholesterol Metabolism by Cytochrome P450scc Show Anti-Leukemia Ef-fects, Having Low or Absent Calcemic Activity. PLoS ONE.

[21] Slominski A.T., Li W., Kim T.K., Semak I., Wang J., Zjawiony J.K., Tuckey R.C. (2015). Novel activities of CYP11A1 and their potential physiological significance. J. Steroid. Biochem. Mol. Biol..

[22] Slominski A.T., Kim T.K., Shehabi H.Z., Semak I., Tang E.K., Nguyen M.N., Benson H.A., Korik E., Janjetovic Z., Chen J. (2012). In vivo evidence for a novel pathway of vitamin D₃ metabolism initiated by P450scc and modified by CYP27B1. FASEB J..

[23] Slominski A.T., Kim T.K., Li W., Postlethwaite A., Tieu E.W., Tang E.K.Y., Tuckey R.C. (2015). Detection of novel CYP11A1-derived secosteroids in the human epidermis and serum and pig adrenal gland. Sci. Rep..

[24] Slominski A.T., Chaiprasongsuk A., Janjetovic Z., Kim T.K., Stefan J., Slominski R.M., Hanumanthu V.S., Raman C., Qayyum S., Song Y. (2020). Photoprotective Properties of Vitamin D and Lumisterol Hydroxyderivatives. Cell Biochem. Biophys..

[25] Slominski A.T., Kim T.K., Takeda Y., Janjetovic Z., Brozyna A.A., Skobowiat C., Wang J., Postlethwaite A., Li W., Tuckey R.C. (2014). RORα and ROR γ are expressed in human skin and serve as receptors for endogenously produced noncalce-mic 20-hydroxy- and 20,23-dihydroxyvitamin D. FASEB J..

[26] Slominski A.T., Kim T.K., Janjetovic Z., Brożyna A.A., Żmijewski M.A., Xu H., Sutter T.R., Tuckey R.C., Jetten A.M., Crossman D.K. (2018). Differential and Overlapping Effects of 20,23(OH)₂D3 and 1,25(OH)₂D3 on Gene Expression in Human Epidermal Keratinocytes: Identification of AhR as an Alternative Receptor for 20,23(OH)₂D3. Int. J. Mol. Sci..

[27] Slominski A.T., Kim T.K., Qayyum S., Song Y., Janjetovic Z., Oak A.S.W., Slominski R.M., Raman C., Stefan J., Mier-Aguilar C.A. (2021). Vitamin D and lumisterol derivatives can act on liver X receptors (LXRs). Sci. Rep..

[28] Agoro R., Ni P., Noonan M.L., White K.E. (2020). Osteocytic FGF23 and Its Kidney Function. Front. Endocrinol..

[29] Tanaka H., Seino Y. (2004). Direct Action of 1,25-Dihydroxyvitamin D on Bone: VDRKO Bone Shows Excessive Bone Formation in Normal Mineral Condition. J. Steroid Biochem. Mol. Biol..

[30] Weissen-Plenz G., Nitschke Y., Rutsch F. (2008). Mechanisms of Arterial Calcification. Spotlight on The Inhibitors. Adv. Clin. Chem..

[31] Sroga G.E., Karim L., Colón W., Vashishth D. (2011). Biochemical Characterization of Major Bone-Matrix Proteins Using Nanoscale-Size Bone Samples and Proteomics Methodology. Mol. Cell Proteom..

[32] Diaz De Barboza G., Guizzardi S., Tolosa De Talamoni N. (2015). Molecular Aspects of Intestinal Calcium Absorption. World J. Gastroenterol..

[33] Haussler M.R., Whitfield G.K., Kaneko I., Haussler C.A., Hsieh D., Hsieh J.C., Jurutka P.W. (2013). Molecular Mechanisms of Vitamin D Action. Calcif. Tissue Int..

[34] Sempos C.T., Vesper H.W., Phinney K.W., Thienpont L.M., Coates P.M. (2012). Vitamin D Status as an International Issue: National Surveys and the Problem of Standardization. Scand. J. Clin. Lab. Investig..

[35] Cashman K.D., Dowling K.G., Škrabáková Z., Gonzalez-Gross M., Valtueña J., de Henauw S., Moreno L., Damsgaard C.T., Michaelsen K.F., Mølgaard C. (2016). Vitamin D Deficiency in Europe: Pandemic?. Am. J. Clin. Nutr..

[36] Schleicher R.L., Sternberg M.R., Looker A.C., Yetley E.A., Lacher D.A., Sempos C.T., Taylor C.L., Durazo-Arvizu R.A., Maw K.L., Chaudhary-Webb M. (2016). National Estimates of Serum Total 25-Hydroxyvitamin D and Metabolite Concentrations Measured by Liquid Chromatography-Tandem Mass Spectrometry in the US Population during 2007–2010. J. Nutr..

[37] Sarafin K., Durazo-Arvizu R., Tian L., Phinney K.W., Tai S., Camara J.E., Merkel J., Green E., Sempos C.T., Brooks S.P.J. (2015). Standardizing 25-Hydroxyvitamin D Values from the Canadian Health Measures Survey. Am. J. Clin. Nutr..

[38] Rabenberg M., Scheidt-Nave C., Busch M.A., Thamm M., Rieckmann N., Durazo-Arvizu R.A., Dowling K.G., Škrabáková Z., Cashman K.D., Sempos C.T. (2018). Implications of Standardization of Serum 25-Hydroxyvitamin D Data for the Evaluation of Vitamin D Status in Germany, Including a Temporal Analysis. BMC Public Health.

[39] Cashman K.D., Dowling K.G., Škrabáková Z., Kiely M., Lamberg-Allardt C., Durazo-Arvizu R.A., Sempos C.T., Koskinen S., Lundqvist A., Sundvall J. (2015). Standardizing Serum 25-Hydroxyvitamin D Data from Four Nordic Population Samples Using the Vitamin D Standardization Program Protocols: Shedding New Light on Vitamin D Status in Nordic Individuals. Scand. J. Clin. Lab. Investig..

[40] Bouillon R. (2017). Comparative Analysis of Nutritional Guidelines for Vitamin D. Nat. Rev. Endocrinol..

[41] Pilz S., März W., Cashman K.D., Kiely M.E., Whiting S.J., Holick M.F., Grant W.B., Pludowski P., Hiligsmann M., Trummer C. (2018). Rationale and Plan for Vitamin D Food Fortification: A Review and Guidance Paper. Front. Endocrinol..

[42] Cashman K.D. (2018). Vitamin D Requirements for the Future—Lessons Learned and Charting a Path Forward. Nutrients.

[43] Pilz S., Trummer C., Pandis M., Schwetz V., Aberer F., Grübler M., Verheyen N., Tomaschitz A., März W. (2018). Vitamin D: Current Guidelines and Future Outlook. Anticancer Res..

[44] Brouwer-Brolsma E.M., Bischoff-Ferrari H.A., Bouillon R., Feskens E.J.M., Gallagher C.J., Hypponen E., Llewellyn D.J., Stoecklin E., Dierkes J., Kies A.K. (2013). Vitamin D: Do We Get Enough?: A Discussion between Vitamin D Experts in Order to Make a Step towards the Harmonisation of Dietary Reference Intakes for Vitamin D across Europe. Osteoporos. Int..

[45] Grossman D.C., Curry S.J., Owens D.K., Barry M.J., Caughey A.B., Davidson K.W., Doubeni C.A., Epling J.W., Kemper A.R., Krist A.H. (2018). Vitamin D, Calcium, OR Combined Supplementation for the Primary Prevention of Fractures in Community-Dwelling Adults Us Preventive Services Task Force Recommendation Statement. JAMA.

[46] Pilz S., Zittermann A., Trummer C., Theiler-Schwetz V., Lerchbaum E., Keppel M.H., Grübler M.R., März W., Pandis M. (2019). Vitamin D Testing and Treatment: A Narrative Review of Current Evidence. Endocr. Connect..

[47] Jääskeläinen T., Itkonen S.T., Lundqvist A., Erkkola M., Koskela T., Lakkala K., Dowling K.G., Hull G.L.J., Kröger H., Karppinen J. (2017). The Positive Impact of General Vitamin D Food Fortification Policy on Vitamin D Status in a Representative Adult Finnish Population: Evidence from an 11-y Follow-up Based on Standardized 25-HydroxyVitamin D Data. Am. J. Clin. Nutr..

[48] Calvo M.S., Whiting S.J. (2013). Survey of Current Vitamin D Food Fortification Practices in the United States and Canada. J. Steroid Biochem. Mol. Biol..

[49] Wilson L.R., Tripkovic L., Hart K.H., Lanham-New S.A. (2017). Vitamin D Deficiency as a Public Health Issue: Using Vitamin D2 or Vitamin D3 in Future Fortification Strategies. Proc. Nutr. Soc..

[50] Moulas A.N., Vaiou M. (2018). Vitamin D Fortification of Foods and Prospective Health Outcomes. J. Biotechnol..

[51] Itkonen S.T., Erkkola M., Lamberg-Allardt C.J.E. (2018). Vitamin D Fortification of Fluid Milk Products and Their Contribution to Vitamin D Intake and Vitamin D Status in Observational Studies—A Review. Nutrients.

[52] Sales N.M.R., Pelegrini P.B., Goersch M.C. (2014). Nutrigenomics: Definitions and Advances of This New Science. J. Nutr. Metab..

[53] Pike J.W. (2011). Genome-wide Principles of Gene Regulation by the Vitamin D Receptor and Its Activating Ligand. Mol. Cell Endocrinol..

[54] Carlberg C. (2019). Nutrigenomics of Vitamin D. Nutrients.

[55] Brumbaugh P.F., Haussler M.R. (1974). 1 Alpha,25-Dihydroxycholecalciferol Receptors in Intestine. I. Association of 1 Alpha,25-Dihydroxycholecalciferol with Intestinal Mucosa Chromatin. J. Biol. Chem..

[56] Pike J.W. (1991). Vitamin D3 Receptors: Structure and Function in Transcription. Annu. Rev. Nutr..

[57] Pike J.W., Meyer M.B., Benkusky N.A., Lee S.M., St. John H., Carlson A., Onal M., Shamsuzzaman S. (2016). Genomic Determinants of Vitamin D-Regulated Gene Expression. Vitam. Horm..

[58] Prüfer K., Racz A., Lin G.C., Barsony J. (2000). Dimerization with Retinoid X Receptors Promotes Nuclear Localization and Subnuclear Targeting of Vitamin D Receptors. J. Biol. Chem..

[59] Pike J.W., Meyer M.B. (2014). Fundamentals of Vitamin D Hormone-Regulated Gene Expression. J. Steroid Biochem. Mol. Biol..

[60] Pike J.W., Meyer M.B., Martowicz M.L., Bishop K.A., Lee S.M., Nerenz R.D., Goetsch P.D. (2010). Emerging Regulatory Paradigms for Control of Gene Expression by 1,25-Dihydroxyvitamin D3. J. Steroid Biochem. Mol. Biol..

[61] Schräder M., Bendik I., Becker-André M., Carlberg C. (1993). Interaction between Retinoic Acid and Vitamin D Signaling Pathways. J. Biol. Chem..

[62] Carlberg C. (2022). Vitamin D and Its Target Genes. Nutrients.

[63] Carlberg C., Molnár F. (2015). Vitamin D Receptor Signaling and Its Therapeutic Implications: Genome-Wide and Structural View. Can. J. Physiol. Pharmacol..

[64] Nandi S., Blais A., Ioshikhes I. (2013). Identification of cis-regulatory modules in promoters of human genes exploiting mutual positioning of transcription factors. Nucleic Acids Res..

[65] Pike J.W., Christakos S. (2017). Biology and Mechanisms of Action of the Vitamin D Hormone. Endocrinol. Metab. Clin. N. Am..

[66] Joshi S., Pantalena L.-C., Liu X.K., Gaffen S.L., Liu H., Rohowsky-Kochan C., Ichiyama K., Yoshimura A., Steinman L., Christakos S. (2011). 1,25-Dihydroxyvitamin D(3) Ameliorates Th17 Autoimmunity via Transcriptional Modulation of Interleukin-17A. Mol. Cell Biol..

[67] Nanduri R., Mahajan S., Bhagyaraj E., Sethi K., Kalra R., Chandra V., Gupta P. (2015). The Active Form of Vitamin D Transcriptionally Represses Smad7 Signaling and Activates Extracellular Signal-Regulated Kinase (ERK) to Inhibit the Differentiation of a Inflammatory T Helper Cell Subset and Suppress Experimental Autoimmune Encephalomyelitis. J. Biol. Chem..

[68] Griffin M.D., Lutz W., Phan V.A., Bachman L.A., McKean D.J., Kumar R. (2001). Dendritic Cell Modulation by 1alpha,25 Dihydroxyvitamin D3 and Its Analogs: A Vitamin D Receptor-Dependent Pathway That Promotes a Persistent State of Immaturity in Vitro and in Vivo. Proc. Natl. Acad. Sci. USA.

[69] Carlberg C. (2017). Molecular Endocrinology of Vitamin D on the Epigenome Level. Mol. Cell Endocrinol..

[70] Fetahu I.S., Höbaus J., Kállay E. (2014). Vitamin D and the Epigenome. Front. Physiol..

[71] Kim S., Shevde N.K., Pike J.W. (2005). 1,25-Dihydroxyvitamin D3 Stimulates Cyclic Vitamin D Receptor/Retinoid X Receptor DNA-Binding, Co-Activator Recruitment, and Histone Acetylation in Intact Osteoblasts. J. Bone Miner. Res..

[72] Nurminen V., Neme A., Seuter S., Carlberg C. (2018). The Impact of the Vitamin D-Modulated Epigenome on VDR Target Gene Regulation. Biochim. Biophys. Acta Gene Regul. Mech..

[73] Herdick M., Carlberg C. (2000). Agonist-Triggered Modulation of the Activated and Silent State of the Vitamin D(3) Receptor by Interaction with Co-Repressors and Co-Activators. J. Mol. Biol..

[74] Polly P., Herdick M., Moehren U., Baniahmad T., Carlberg C. (2000). VDR-Alien: A Novel, DNA-Selective Vitamin D(3) Receptor-Corepressor Partnership. FASEB J..

[75] Pereira F., Barbáchano A., Silva J., Bonilla F., Campbell M.J., Muñoz A., Larriba M.J. (2011). KDM6B/JMJD3 Histone Demethylase Is Induced by Vitamin D and Modulates Its Effects in Colon Cancer Cells. Hum. Mol. Genet..

[76] Battaglia S., Karasik E., Gillard B., Williams J., Winchester T., Moser M.T., Smiraglia D.J., Foster B.A. (2017). LSD1 Dual Function in Mediating Epigenetic Corruption of the Vitamin D Signaling in Prostate Cancer. Clin. Epigenet..

[77] Wei Z., Yoshihara E., He N., Hah N., Fan W., Pinto A.F.M., Huddy T., Wang Y., Ross B., Estepa G. (2018). Vitamin D Switches BAF Complexes to Protect β Cells. Cell.

[78] Prietl B., Treiber G., Pieber T.R., Amrein K. (2013). Vitamin D and Immune Function. Nutrients.

[79] von Essen M.R., Kongsbak M., Schjerling P., Olgaard K., Ødum N., Geisler C. (2010). Vitamin D Controls T Cell Antigen Receptor Signaling and Activation of Human T Cells. Nat. Immunol..

[80] Heine G., Niesner U., Chang H.D., Steinmeyer A., Zügel U., Zuberbier T., Radbruch A., Worm M. (2008). 1,25-Dihydroxyvitamin D(3) Promotes IL-10 Production in Human B Cells. Eur. J. Immunol..

[81] Jeffery L.E., Wood A.M., Qureshi O.S., Hou T.Z., Gardner D., Briggs Z., Kaur S., Raza K., Sansom D.M. (2012). Availability of 25-Hydroxyvitamin D(3) to APCs Controls the Balance between Regulatory and Inflammatory T Cell Responses. J. Immunol..

[82] Sigmundsdottir H., Pan J., Debes G.F., Alt C., Habtezion A., Soler D., Butcher E.C. (2007). DCs Metabolize Sunlight-Induced Vitamin D3 to “program” T Cell Attraction to the Epidermal Chemokine CCL27. Nat. Immunol..

[83] Kongsbak M., von Essen M.R., Levring T.B., Schjerling P., Woetmann A., Ødum N., Bonefeld C.M., Geisler C. (2014). Vitamin D-Binding Protein Controls T Cell Responses to Vitamin D. BMC Immunol..

[84] Esteban L., Vidal M., Dusso A. (2004). 1alpha-Hydroxylase Transactivation by Gamma-Interferon in Murine Macrophages Requires Enhanced C/EBPbeta Expression and Activation. J. Steroid Biochem. Mol. Biol..

[85] Baeke F., Korf H., Overbergh L., van Etten E., Verstuyf A., Gysemans C., Mathieu C. (2010). Human T Lymphocytes Are Direct Targets of 1,25-Dihydroxyvitamin D3 in the Immune System. J. Steroid Biochem. Mol. Biol..

[86] XU H., SORURI A., GIESELER R.K.H., PETERS J.H. (1993). 1,25-Dihydroxyvitamin D3 Exerts Opposing Effects to IL-4 on MHC Class-II Antigen Expression, Accessory Activity, and Phagocytosis of Human Monocytes. Scand. J. Immunol..

[87] Baeke F., Takiishi T., Korf H., Gysemans C., Mathieu C. (2010). Vitamin D: Modulator of the Immune System. Curr. Opin. Pharmacol..

[88] Wang T.-T., Nestel F.P., Bourdeau V., Nagai Y., Wang Q., Liao J., Tavera-Mendoza L., Lin R., Hanrahan J.W., Mader S. (2004). Cutting Edge: 1,25-Dihydroxyvitamin D3 Is a Direct Inducer of Antimicrobial Peptide Gene Expression. J. Immunol..

[89] Gombart A.F., Borregaard N., Koeffler H.P. (2005). Human Cathelicidin Antimicrobial Peptide (CAMP) Gene Is a Direct Target of the Vitamin D Receptor and Is Strongly up-Regulated in Myeloid Cells by 1,25-Dihydroxyvitamin D3. FASEB J..

[90] White J.H. (2012). Vitamin D Metabolism and Signaling in the Immune System. Rev. Endocr. Metab. Disord..

[91] Zhang Y., Leung D.Y.M., Richers B.N., Liu Y., Remigio L.K., Riches D.W., Goleva E. (2012). Vitamin D Inhibits Monocyte/Macrophage Proinflammatory Cytokine Production by Targeting MAPK Phosphatase-1. J. Immunol..

[92] Wancket L.M., Frazier W.J., Liu Y. (2012). Mitogen-activated protein kinase phosphatase (MKP)-1 in immunology, physiology, and disease. Life Sci..

[93] Wang Q., He Y., Shen Y., Zhang Q., Chen D., Zuo C., Qin J., Wang H., Wang J., Yu Y. (2014). Vitamin D Inhibits COX-2 Expression and Inflammatory Response by Targeting Thioesterase Superfamily Member 4. J. Biol. Chem..

[94] Ferreira G.B., van Etten E., Verstuyf A., Waer M., Overbergh L., Gysemans C., Mathieu C. (2011). 1,25-Dihydroxyvitamin D3 Alters Murine Dendritic Cell Behaviour in Vitro and in Vivo. Diabetes Metab. Res. Rev..

[95] Ao T., Kikuta J., Ishii M. (2021). The Effects of Vitamin D on Immune System and Inflammatory Diseases. Biomolecules.

[96] Sommer A., Fabri M. (2015). Vitamin D regulates cytokine patterns secreted by dendritic cells to promote differentiation of IL-22-producing T cells. PLoS One.

[97] Penna G., Amuchastegui S., Giarratana N., Daniel K.C., Vulcano M., Sozzani S., Adorini L. (2007). 1,25-Dihydroxyvitamin D3 Selectively Modulates Tolerogenic Properties in Myeloid but Not Plasmacytoid Dendritic Cells. J. Immunol..

[98] Veldman C.M., Cantorna M.T., DeLuca H.F. (2000). Expression of 1,25-Dihydroxyvitamin D(3) Receptor in the Immune System. Arch. Biochem. Biophys..

[99] Ahangar-Parvin R., Mohammadi-Kordkhayli M., Azizi S.V., Nemati M., Khorramdelazad H., Taghipour Z., Hassan Z., Moazzeni S.M., Jafarzadeh A. (2018). The Modulatory Effects of Vitamin D on the Expression of IL-12 and TGF-β in the Spinal Cord and Serum of Mice with Experimental Autoimmune Encephalomyelitis. Iran. J. Pathol..

[100] Martens P.J., Gysemans C., Verstuyf A., Mathieu C. (2020). Vitamin D’s Effect on Immune Function. Nutrients.

[101] Agraz-Cibrian J.M., Giraldo D.M., Urcuqui-Inchima S. (2019). 1,25-Dihydroxyvitamin D 3 Induces Formation of Neutrophil Extracellular Trap-like Structures and Modulates the Transcription of Genes Whose Products Are Neutrophil Extracellular Trap-Associated Proteins: A Pilot Study. Steroids.

[102] Takahashi K., Nakayama Y., Horiuchi H., Ohta T., Komoriya K., Ohmori H., Kamimura T. (2002). Human Neutrophils Express Messenger RNA of Vitamin D Receptor and Respond to 1alpha,25-Dihydroxyvitamin D3. Immunopharmacol. Immunotoxicol..

[103] Mora J.R., Iwata M., von Andrian U.H. (2008). Vitamin Effects on the Immune System: Vitamins A and D Take Centre Stage. Nat. Rev. Immunol..

[104] Chen S., Sims G.P., Chen X.X., Gu Y.Y., Chen S., Lipsky P.E. (2007). Modulatory Effects of 1,25-Dihydroxyvitamin D3 on Human B Cell Differentiation. J. Immunol..

[105] Drozdenko G., Scheel T., Heine G., Baumgrass R., Worm M. (2014). Impaired T Cell Activation and Cytokine Production by Calcitriol-Primed Human B Cells. Clin. Exp. Immunol..

[106] Danner O.K., Matthews L.R., Francis S., Rao V.N., Harvey C.P., Tobin R.P., Wilson K.L., Alema-Mensah E., Newell Rogers M.K., Childs E.W. (2016). Vitamin D3 Suppresses Class II Invariant Chain Peptide Expression on Activated B-Lymphocytes: A Plausible Mechanism for Downregulation of Acute Inflammatory Conditions. J. Nutr. Metab..

[107] Colotta F., Jansson B., Bonelli F. (2017). Modulation of Inflammatory and Immune Responses by Vitamin D. J. Autoimmun..

[108] Lemire J.M., Adams J.S., Kermani-Arab V., Bakke A.C., Sakai R., Jordan S.C. (1985). 1,25-Dihydroxyvitamin D3 suppresses human T helper/inducer lymphocyte activity in vitro. J. Immunol..

[109] Cantorna M.T. (2010). Mechanisms Underlying the Effect of Vitamin D on the Immune System. Proc. Nutr. Soc..

[110] Baeke F., Korf H., Overbergh L., Verstuyf A., Thorrez L., van Lommel L., Waer M., Schuit F., Gysemans C., Mathieu C. (2011). The Vitamin D Analog, TX527, Promotes a Human CD4+CD25highCD127low Regulatory T Cell Profile and Induces a Migratory Signature Specific for Homing to Sites of Inflammation. J. Immunol..

[111] Lemire J.M., Archer D.C., Beck L., Spiegelberg H.L. (1995). Immunosuppressive actions of 1,25-dihydroxyvitamin D3: Preferential inhibition of Th1 functions. J. Nutr..

[112] van Belle T.L., Gysemans C., Mathieu C. (2011). Vitamin D in Autoimmune, Infectious and Allergic Diseases: A Vital Player?. Best Pract. Res. Clin. Endocrinol. Metab..

[113] Palmer M.T., Lee Y.K., Maynard C.L., Oliver J.R., Bikle D.D., Jetten A.M., Weaver C.T. (2011). Lineage-Specific Effects of 1,25-Dihydroxyvitamin D(3) on the Development of Effector CD4 T Cells. J. Biol. Chem..

[114] Giulietti A., Gysemans C., Stoffels K., van Etten E., Decallonne B., Overbergh L., Bouillon R., Mathieu C. (2004). Vitamin D Deficiency in Early Life Accelerates Type 1 Diabetes in Non-Obese Diabetic Mice. Diabetologia.

[115] da Costa D.S., Hygino J., Ferreira T.B., Kasahara T.M., Barros P.O., Monteiro C., Oliveira A., Tavares F., Vasconcelos C.C., Alvarenga R. (2016). Vitamin D modulates different IL-17-secreting T cell subsets in multiple sclerosis patients. J. Neuroimmunol..

[116] Boonstra A., Barrat F.J., Crain C., Heath V.L., Savelkoul H.F.J., O’Garra A. (2001). 1alpha,25-Dihydroxyvitamin D3 Has a Direct Effect on Naive CD4(+) T Cells to Enhance the Development of Th2 Cells. J. Immunol..

[117] Lysandropoulos A.P., Jaquiéry E., Jilek S., Pantaleo G., Schluep M., du Pasquier R.A. (2011). Vitamin D Has a Direct Immunomodulatory Effect on CD8+ T Cells of Patients with Early Multiple Sclerosis and Healthy Control Subjects. J. Neuroimmunol..

[118] Aly M.G., Trojan K., Weimer R., Morath C., Opelz G., Tohamy M.A., Daniel V. (2016). Low-Dose Oral Cholecalciferol Is Associated with Higher Numbers of Helios(+) and Total Tregs than Oral Calcitriol in Renal Allograft Recipients: An Observational Study. BMC Pharmacol. Toxicol..

[119] Bock G., Prietl B., Mader J.K., Höller E., Wolf M., Pilz S., Graninger W.B., Obermayer-Pietsch B.M., Pieber T.R. (2011). The Effect of Vitamin D Supplementation on Peripheral Regulatory T Cells and β Cell Function in Healthy Humans: A Randomized Controlled Trial. Diabetes Metab. Res. Rev..

[120] Chen L., Cencioni M.T., Angelini D.F., Borsellino G., Battistini L., Brosnan C.F. (2005). Transcriptional Profiling of Gamma Delta T Cells Identifies a Role for Vitamin D in the Immunoregulation of the V Gamma 9V Delta 2 Response to Phosphate-Containing Ligands. J. Immunol..

[121] Waddell A., Zhao J., Cantorna M.T. (2015). NKT Cells Can Help Mediate the Protective Effects of 1,25-Dihydroxyvitamin D3 in Experimental Autoimmune Encephalomyelitis in Mice. Int. Immunol..

[122] Mazahery H., von Hurst P.R. (2015). Factors Affecting 25-Hydroxyvitamin D Concentration in Response to Vitamin D Supplementation. Nutrients.

[123] (2021). 2021 Alzheimer’s Disease Facts and Figures. Alzheimer’s Dement..

[124] Jack C.R., Bennett D.A., Blennow K., Carrillo M.C., Dunn B., Haeberlein S.B., Holtzman D.M., Jagust W., Jessen F., Karlawish J. (2018). NIA-AA Research Framework: Toward a Biological Definition of Alzheimer’s Disease. Alzheimer’s Dement..

[125] Hardy J., Selkoe D.J. (2002). The Amyloid Hypothesis of Alzheimer’s Disease: Progress and Problems on the Road to Therapeutics. Science.

[126] Ahmed Z., Cooper J., Murray T.K., Garn K., McNaughton E., Clarke H., Parhizkar S., Ward M.A., Cavallini A., Jackson S. (2014). A Novel in Vivo Model of Tau Propagation with Rapid and Progressive Neurofibrillary Tangle Pathology: The Pattern of Spread Is Determined by Connectivity, Not Proximity. Acta Neuropathol..

[127] Sonawane S.K., Chinnathambi S. (2018). Prion-Like Propagation of Post-Translationally Modified Tau in Alzheimer’s Disease: A Hypothesis. J. Mol. Neurosci..

[128] Bhatia S., Rawal R., Sharma P., Singh T., Singh M., Singh V. (2021). Mitochondrial Dysfunction in Alzheimer’s Disease: Opportunities for Drug Development. Curr. Neuropharmacol..

[129] Halliday G., Robinson S.R., Shepherd C., Kril J. (2000). Alzheimer’s Disease and Inflammation: A Review of Cellular and Therapeutic Mechanisms. Clin. Exp. Pharmacol. Physiol..

[130] Mentis A.F.A., Dardiotis E., Chrousos G.P. (2021). Apolipoprotein E4 and Meningeal Lymphatics in Alzheimer Disease: A Conceptual Framework. Mol. Psychiatry.

[131] Zatta P., Drago D., Bolognin S., Sensi S.L. (2009). Alzheimer’s Disease, Metal Ions and Metal Homeostatic Therapy. Trends Pharmacol. Sci..

[132] Sweeney M.D., Montagne A., Sagare A.P., Nation D.A., Schneider L.S., Chui H.C., Harrington M.G., Pa J., Law M., Wang D.J.J. (2019). Vascular Dysfunction—The Disregarded Partner of Alzheimer’s Disease. Alzheimers Dement..

[133] Popugaeva E., Pchitskaya E., Bezprozvanny I. (2017). Dysregulation of Neuronal Calcium Homeostasis in Alzheimer’s Disease—A Therapeutic Opportunity?. Biochem. Biophys. Res. Commun..

[134] Wong K.Y., Roy J., Fung M.L., Heng B.C., Zhang C., Lim L.W. (2020). Relationships between Mitochondrial Dysfunction and Neurotransmission Failure in Alzheimer’s Disease. Aging Dis..

[135] Tönnies E., Trushina E. (2017). Oxidative Stress, Synaptic Dysfunction, and Alzheimer’s Disease. J. Alzheimers Dis..

[136] Cai Q., Tammineni P. (2017). Mitochondrial Aspects of Synaptic Dysfunction in Alzheimer’s Disease. J. Alzheimers Dis..

[137] Masoumi A., Goldenson B., Ghirmai S., Avagyan H., Zaghi J., Abel K., Zheng X., Espinosa-Jeffrey A., Mahanian M., Liu P.T. (2009). 1α,25-Dihydroxyvitamin D3 Interacts with Curcuminoids to Stimulate Amyloid-β Clearance by Macrophages of Alzheimer’s Disease Patients. J. Alzheimers Dis..

[138] Patel P., Shah J. (2017). Role of Vitamin D in Amyloid Clearance via LRP-1 Upregulation in Alzheimer’s Disease: A Potential Therapeutic Target?. J. Chem. Neuroanat..

[139] Mizwicki M.T., Menegaz D., Zhang J., Barrientos-Durán A., Tse S., Cashman J.R., Griffin P.R., Fiala M. (2012). Genomic and Nongenomic Signaling Induced by 1α,25(OH) 2-Vitamin D3 Promotes the Recovery of Amyloid-β Phagocytosis by Alzheimer’s Disease Macrophages. J. Alzheimers Dis..

[140] Burton T., Liang B., Dibrov A., Amara F. (2002). Transforming Growth Factor-β-Induced Transcription of the Alzheimer β-Amyloid Precursor Protein Gene Involves Interaction between the CTCF-Complex and Smads. Biochem. Biophys. Res. Commun..

[141] Yanagisawa J., Yanagi Y., Masuhiro Y., Suzawa M., Watanabe M., Kashiwagi K., Toriyabe T., Kawabata M., Miyazono K., Kato S. (1999). Convergence of Transforming Growth Factor-β and Vitamin D Signaling Pathways on SMAD Transcriptional Coactivators. Science.

[142] Wimalawansa S.J. (2019). Vitamin D Deficiency: Effects on Oxidative Stress, Epigenetics, Gene Regulation, and Aging. Biology.

[143] Bivona G., Lo Sasso B., Gambino C.M., Giglio R.V., Scazzone C., Agnello L., Ciaccio M. (2021). The Role of Vitamin D as a Biomarker in Alzheimer’s Disease. Brain Sci..

[144] Banerjee A., Khemka V.K., Ganguly A., Roy D., Ganguly U., Chakrabarti S. (2015). Vitamin D and Alzheimer’s Disease: Neurocognition to Therapeutics. Int. J. Alzheimers Dis..

[145] Littlejohns T.J., Henley W.E., Lang I.A., Annweiler C., Beauchet O., Chaves P.H.M., Fried L., Kestenbaum B.R., Kuller L.H., Langa K.M. (2014). Vitamin D and the Risk of Dementia and Alzheimer Disease. Neurology.

[146] Licher S., de Bruijn R.F.A.G., Wolters F.J., Zillikens M.C., Ikram M.A., Ikram M.K. (2017). Vitamin D and the Risk of Dementia: The Rotterdam Study. J. Alzheimers Dis..

[147] Buell J.S., Dawson-Hughes B., Scott T.M., Weiner D.E., Dallal G.E., Qui W.Q., Bergethon P., Rosenberg I.H., Folstein M.F., Patz S. (2010). 25-Hydroxyvitamin D, Dementia, and Cerebrovascular Pathology in Elders Receiving Home Services. Neurology.

[148] Afzal S., Bojesen S.E., Nordestgaard B.G. (2014). Reduced 25-Hydroxyvitamin D and Risk of Alzheimer’s Disease and Vascular Dementia. Alzheimers Dement..

[149] Etgen T., Sander D., Bickel H., Sander K., Förstl H. (2012). Vitamin D Deficiency, Cognitive Impairment and Dementia: A Systematic Review and Meta-Analysis. Dement. Geriatr. Cogn. Disord..

[150] Knekt P., Sääksjärvi K., Järvinen R., Marniemi J., Männistö S., Kanerva N., Heliövaara M. (2014). Serum 25-Hydroxyvitamin D Concentration and Risk of Dementia. Epidemiology.

[151] Balion C., Griffith L.E., Strifler L., Henderson M., Patterson C., Heckman G., Llewellyn D.J., Raina P. (2012). Vitamin D, Cognition, and Dementia; A Systematic Review and Meta-Analysis. Neurology.

[152] Feart C., Helmer C., Merle B., Herrmann F.R., Annweiler C., Dartigues J.F., Delcourt C., Samieri C. (2017). Associations of Lower Vitamin D Concentrations with Cognitive Decline and Long-Term Risk of Dementia and Alzheimer’s Disease in Older Adults. Alzheimers Dement..

[153] Ouma S., Suenaga M., Bölükbaşı Hatip F.F., Hatip-Al-Khatib I., Tsuboi Y., Matsunaga Y. (2018). Serum Vitamin D in Patients with Mild Cognitive Impairment and Alzheimer’s Disease. Brain Behav..

[154] Wang L., Qiao Y., Zhang H., Zhang Y., Hua J., Jin S., Liu G. (2020). Circulating Vitamin D Levels and Alzheimer’s Disease: A Mendelian Randomization Study in the IGAP and UK Biobank. J. Alzheimers Dis..

[155] Ulstein I., Bohmer T. (2017). Normal Vitamin Levels and Nutritional Indices in Alzheimer’s Disease Patients with Mild Cognitive Impairment or Dementia with Normal Body Mass Indexes. J. Alzheimers Dis..

[156] Karakis I., Pase M.P., Beiser A., Booth S.L., Jacques P.F., Rogers G., DeCarli C., Vasan R.S., Wang T.J., Himali J.J. (2016). Association of Serum Vitamin D with the Risk of Incident Dementia and Subclinical Indices of Brain Aging: The Framingham Heart Study. J. Alzheimers Dis..

[157] Yang K., Chen J., Li X., Zhou Y. (2019). Vitamin D Concentration and Risk of Alzheimer Disease: A Meta-Analysis of Prospective Cohort Studies. Medicine.

[158] Jayedi A., Rashidy-Pour A., Shab-Bidar S. (2019). Vitamin D status and risk of dementia and Alzheimer’s disease: A meta-analysis of dose-response. Nutr. Neurosci..

[159] Chai B., Gao F., Wu R., Dong T., Gu C., Lin Q., Zhang Y. (2019). Vitamin D deficiency as a risk factor for dementia and Alzheimer’s disease: An updated meta-analysis. BMC Neurol..

[160] Du Y., Liang F., Zhang L., Liu J., Dou H. (2020). Vitamin D Supplement for Prevention of Alzheimer’s Disease: A Systematic Review and Meta-Analysis. Am. J. Ther..

[161] Bode L.E., McClester Brown M., Hawes E.M. (2020). Vitamin D Supplementation for Extraskeletal Indications in Older Persons. J. Am. Med. Dir. Assoc..

[162] Rutjes A.W.S., Denton D.A., di Nisio M., Chong L.Y., Abraham R.P., Al-Assaf A.S., Anderson J.L., Malik M.A., Vernooij R.W.M., Martínez G. (2018). Vitamin and Mineral Supplementation for Maintaining Cognitive Function in Cognitively Healthy People in Mid and Late Life. Cochrane Database Syst. Rev..

[163] Jorde R., Kubiak J., Svartberg J., Fuskevåg O.M., Figenschau Y., Martinaityte I., Grimnes G. (2019). Vitamin D Supplementation Has No Effect on Cognitive Performance after Four Months in Mid-Aged and Older Subjects. J. Neurol. Sci..

[164] Bischoff-Ferrari H.A., Vellas B., Rizzoli R., Kressig R.W., da Silva J.A.P., Blauth M., Felson D.T., McCloskey E.V., Watzl B., Hofbauer L.C. (2020). Effect of Vitamin D Supplementation, Omega-3 Fatty Acid Supplementation, or a Strength-Training Exercise Program on Clinical Outcomes in Older Adults: The DO-HEALTH Randomized Clinical Trial. JAMA.

[165] Moran C., Scotto di Palumbo A., Bramham J., Moran A., Rooney B., de Vito G., Egan B. (2018). Effects of a Six-Month Multi-Ingredient Nutrition Supplement Intervention of Omega-3 Polyunsaturated Fatty Acids, Vitamin D, Resveratrol, and Whey Protein on Cognitive Function in Older Adults: A Randomised, Double-Blind, Controlled Trial. J. Prev. Alzheimers Dis..

[166] Gil Martínez V., Avedillo Salas A., Santander Ballestín S. (2022). Vitamin Supplementation and Dementia: A Systematic Review. Nutrients.

[167] Przybelski R., Agrawal S., Krueger D., Engelke J.A., Walbrun F., Binkley N. (2008). Rapid Correction of Low Vitamin D Status in Nursing Home Residents. Osteoporos. Int..

[168] Stein M.S., Scherer S.C., Ladd K.S., Harrison L.C. (2011). A Randomized Controlled Trial of High-Dose Vitamin D2 Followed by Intranasal Insulin in Alzheimer’s Disease. J. Alzheimers Dis..

[169] Jia J., Hu J., Huo X., Miao R., Zhang Y., Ma F. (2019). Effects of Vitamin D Supplementation on Cognitive Function and Blood Aβ-Related Biomarkers in Older Adults with Alzheimer’s Disease: A Randomised, Double-Blind, Placebo-Controlled Trial. J. Neurol. Neurosurg. Psychiatry.

[170] Yang T., Wang H., Xiong Y., Chen C., Duan K., Jia J., Ma F. (2020). Vitamin D Supplementation Improves Cognitive Function through Reducing Oxidative Stress Regulated by Telomere Length in Older Adults with Mild Cognitive Impairment: A 12-Month Randomized Controlled Trial. J. Alzheimers Dis..

[171] Lai R.H., Hsu C.C., Yu B.H., Lo Y.R., Hsu Y.Y., Chen M.H., Juang J.L. (2022). Vitamin D supplementation worsens Alzheimer’s progression: Animal model and human cohort studies. Aging Cell.

[172] Poewe W., Seppi K., Tanner C.M., Halliday G.M., Brundin P., Volkmann J., Schrag A.E., Lang A.E. (2017). Parkinson Disease. Nat. Rev. Dis. Prim..

[173] Samuel S., Sitrin M.D. (2008). Vitamin D’s Role in Cell Proliferation and Differentiation. Nutr. Rev..

[174] Knekt P., Kilkkinen A., Rissanen H., Marniemi J., Sääksjärvi K., Heliövaara M. (2010). Serum Vitamin D and the Risk of Parkinson Disease. Arch. Neurol..

[175] Shrestha S., Lutsey P.L., Alonso A., Huang X., Mosley T.H., Chen H. (2016). Serum 25-Hydroxyvitamin D Concentrations in Mid-Adulthood and Parkinson’s Disease Risk. Mov. Disord..

[176] Fullard M.E., Xie S.X., Marek K., Stern M., Jennings D., Siderowf A., Willis A.W., Chen-Plotkin A.S. (2017). Vitamin D in the Parkinson Associated Risk Syndrome (PARS) Study. Mov. Disord..

[177] Wang X., Shen N., Lu Y., Tan K. (2019). Vitamin D Receptor Polymorphisms and the Susceptibility of Parkinson’s Disease. Neurosci. Lett..

[178] Meamar R., Shaabani P., Tabibian S.R., Aghaye Ghazvini M.R., Feizi A. (2015). The Effects of Uric Acid, Serum Vitamin D3, and Their Interaction on Parkinson’s Disease Severity. Park. Dis..

[179] Luo X., Ou R., Dutta R., Tian Y., Xiong H., Shang H. (2018). Association between Serum Vitamin D Levels and Parkinson’s Disease: A Systematic Review and Meta-Analysis. Front. Neurol..

[180] Ding H., Dhima K., Lockhart K.C., Locascio J.J., Hoesing A.N., Duong K., Trisini-Lipsanopoulos A., Hayes M.T., Sohur U.S., Wills A.M. (2013). Unrecognized Vitamin D3 Deficiency Is Common in Parkinson Disease: Harvard Biomarker Study. Neurology.

[181] Suzuki M., Yoshioka M., Hashimoto M., Murakami M., Kawasaki K., Noya M., Takahashi D., Urashima M. (2012). 25-Hydroxyvitamin D, Vitamin D Receptor Gene Polymorphisms, and Severity of Parkinson’s Disease. Mov. Disord..

[182] Evatt M.L., DeLong M.R., Kumari M., Auinger P., McDermott M.P., Tangpricha V. (2011). High Prevalence of Hypovitaminosis D Status in Patients with Early Parkinson Disease. Arch. Neurol..

[183] McCarty D.E., Reddy A., Keigley Q., Kim P.Y., Marino A.A. (2012). Vitamin D, Race, and Excessive Daytime Sleepiness. J. Clin. Sleep Med..

[184] Kim J.E., Oh E., Park J., Youn J., Kim J.S., Jang W. (2018). Serum 25-Hydroxyvitamin D3 Level May Be Associated with Olfactory Dysfunction in de Novo Parkinson’s Disease. J. Clin. Neurosci..

[185] Peterson A.L., Murchison C., Zabetian C., Leverenz J.B., Watson G.S., Montine T., Carney N., Bowman G.L., Edwards K., Quinn J.F. (2013). Memory, Mood, and Vitamin D in Persons with Parkinson’s Disease. J. Park. Dis..

[186] Wei F.L., Li T., Gao Q.Y., Huang Y., Zhou C.P., Wang W., Qian J.X. (2022). Association Between Vitamin D Supplementation and Fall Prevention. Front. Endocrinol..

[187] Bischoff-Ferrari H.A., Dawson-Hughes B., Willett W.C., Staehelin H.B., Bazemore M.G., Zee R.Y., Wong J.B. (2004). Effect of Vitamin D on falls: A meta-analysis. JAMA.

[188] Gillespie L.D., Robertson M.C., Gillespie W.J., Sherrington C., Gates S., Clemson L.M., Lamb S.E. (2012). Interventions for preventing falls in older people living in the community. Cochrane Database Syst. Rev..

[189] Suzuki M., Yoshioka M., Hashimoto M., Murakami M., Noya M., Takahashi D., Urashima M. (2013). Randomized, Double-Blind, Placebo-Controlled Trial of Vitamin D Supplementation in Parkinson Disease. Am. J. Clin. Nutr..

[190] Luthra N.S., Kim S., Zhang Y., Christine C.W. (2018). Characterization of Vitamin D Supplementation and Clinical Outcomes in a Large Cohort of Early Parkinson’s Disease. J. Clin. Mov. Disord..

[191] Masrori P., van Damme P. (2020). Amyotrophic Lateral Sclerosis: A Clinical Review. Eur. J. Neurol..

[192] Hardiman O., Al-Chalabi A., Chio A., Corr E.M., Logroscino G., Robberecht W., Shaw P.J., Simmons Z., van den Berg L.H. (2017). Amyotrophic Lateral Sclerosis. Nat. Rev. Dis. Prim..

[193] Lanznaster D., Bejan-Angoulvant T., Gandía J., Blasco H., Corcia P. (2020). Is There a Role for Vitamin D in Amyotrophic Lateral Sclerosis? A Systematic Review and Meta-Analysis. Front. Neurol..

[194] Crick P.J., Griffiths W.J., Zhang J., Beibel M., Abdel-Khalik J., Kuhle J., Sailer A.W., Wang Y. (2017). Reduced Plasma Levels of 25-Hydroxycholesterol and Increased Cerebrospinal Fluid Levels of Bile Acid Precursors in Multiple Sclerosis Patients. Mol. Neurobiol..

[195] Elf K., Askmark H., Nygren I., Punga A.R. (2014). Vitamin D Deficiency in Patients with Primary Immune-Mediated Peripheral Neuropathies. J. Neurol. Sci..

[196] Libonati L., Onesti E., Gori M.C., Ceccanti M., Cambieri C., Fabbri A., Frasca V., Inghilleri M. (2017). Vitamin D in Amyotrophic Lateral Sclerosis. Funct. Neurol..

[197] Cortese R., D’Errico E., Introna A., Schirosi G., Scarafino A., Distaso E., Nazzaro P., Zoccolella S., Simone I. (2015). Vitamin D Levels in Serum of Amyotrophic Lateral Sclerosis Patients. (P2.069). Neurology.

[198] Paganoni S., Macklin E.A., Karam C., Yu H., Gonterman F., Fetterman K.A., Cudkowicz M., Berry J., Wills A.M. (2017). Vitamin D Levels Are Associated with Gross Motor Function in Amyotrophic Lateral Sclerosis. Muscle Nerve.

[199] Blasco H., Madji Hounoum B., Dufour-Rainfray D., Patin F., Maillot F., Beltran S., Gordon P.H., Andres C.R., Corcia P. (2015). Vitamin D Is Not a Protective Factor in ALS. CNS Neurosci. Ther..

[200] Camu W., Tremblier B., Plassot C., Alphandery S., Salsac C., Pageot N., Juntas-Morales R., Scamps F., Daures J.P., Raoul C. (2014). Vitamin D Confers Protection to Motoneurons and Is a Prognostic Factor of Amyotrophic Lateral Sclerosis. Neurobiol. Aging.

[201] Yang J., Park J.S., Oh K.W., Oh S., Park H.M., Kim S.H. (2016). Vitamin D Levels Are Not Predictors of Survival in a Clinic Population of Patients with ALS. J. Neurol. Sci..

[202] Juntas-Morales R., Pageot N., Marin G., Dupuy A.M., Alphandery S., Labar L., Esselin F., Picot M.C., Camu W. (2020). Low 25OH Vitamin D Blood Levels Are Independently Associated with Higher Amyotrophic Lateral Sclerosis Severity Scores: Results from a Prospective Study. Front. Neurol..

[203] Török N., Török R., Klivényi P., Engelhardt J., Vécsei L. (2016). Investigation of vitamin D receptor polymorphisms in amyotrophic lateral sclerosis. Acta Neurol. Scand..

[204] Dardiotis E., Siokas V., Sokratous M., Tsouris Z., Michalopoulou A., Andravizou A., Dastamani M., Ralli S., Vinceti M., Tsatsakis A. (2018). Genetic polymorphisms in amyotrophic lateral sclerosis: Evidence for implication in detoxification pathways of environmental toxicants. Environ. Int..

[205] Trojsi F., Siciliano M., Passaniti C., Bisecco A., Russo A., Lavorgna L., Esposito S., Ricciardi D., Monsurrò M.R., Tedeschi G. (2020). Vitamin D Supplementation Has No Effects on Progression of Motor Dysfunction in Amyotrophic Lateral Sclerosis (ALS). Eur. J. Clin. Nutr..

[206] Karam C., Barrett M.J., Imperato T., Macgowan D.J.L., Scelsa S. (2013). Vitamin D Deficiency and Its Supplementation in Patients with Amyotrophic Lateral Sclerosis. J. Clin. Neurosci..

[207] Brownlee W.J., Hardy T.A., Fazekas F., Miller D.H. (2017). Diagnosis of Multiple Sclerosis: Progress and Challenges. Lancet.

[208] Lublin F.D., Reingold S.C., Cohen J.A., Cutter G.R., Sørensen P.S., Thompson A.J., Wolinsky J.S., Balcer L.J., Banwell B., Barkhof F. (2014). Defining the Clinical Course of Multiple Sclerosis: The 2013 Revisions. Neurology.

[209] Miller D.H., Leary S.M. (2007). Primary-Progressive Multiple Sclerosis. Lancet Neurol..

[210] Ascherio A. (2013). Environmental Factors in Multiple Sclerosis. Expert Rev. Neurother..

[211] Vukusic S., van Bockstael V., Gosselin S., Confavreux C. (2007). Regional Variations in the Prevalence of Multiple Sclerosis in French Farmers. J. Neurol. Neurosurg. Psychiatry.

[212] Hammond S.R., Mcleod J.G., Millingen K.S., Stewart-wynne E.G., English D., Holland J.T., Mccall M.G. (1988). The Epidemiology of Multiple Sclerosis in Three Australian Cities: Perth, Newcastle and Hobart. Brain.

[213] Miller D.H., Purdie G., Hammond S.R., McLeod J.G., Skegg D.C.G. (1990). Multiple Sclerosis in Australia and New Zealand: Are the Determinants Genetic or Environmental?. J. Neurol. Neurosurg. Psychiatry.

[214] Acheson E.D., Bachrach C.A., Wright F.M. (1960). Some comments on the relationship of the distribution of multiple sclerosis to latitude, solar radiation, and other variables. Acta Psychiatr. Scand..

[215] Kurtzke J.F., Beebe G.W., Norman J.E. (1979). Epidemiology of Multiple Sclerosis in U.S. Veterans: 1. Race, Sex, and Geographic Distribution. Neurology.

[216] Bäärnhielm M., Olsson T., Alfredsson L. (2014). Fatty Fish Intake Is Associated with Decreased Occurrence of Multiple Sclerosis. Mult. Scler..

[217] Swank R.L., Lerstad O., Strøm A., Backer J. (1952). Multiple sclerosis in rural Norway its geographic and occupational incidence in relation to nutrition. N. Engl. J. Med..

[218] Kampman M.T., Wilsgaard T., Mellgren S.I. (2007). Outdoor Activities and Diet in Childhood and Adolescence Relate to MS Risk above the Arctic Circle. J. Neurol..

[219] Behrens J.R., Rasche L., Gieß R.M., Pfuhl C., Wakonig K., Freitag E., Deuschle K., Bellmann-Strobl J., Paul F., Ruprecht K. (2016). Low 25-Hydroxyvitamin D, but Not the Bioavailable Fraction of 25-Hydroxyvitamin D, Is a Risk Factor for Multiple Sclerosis. Eur. J. Neurol..

[220] Munger K.L., Levin L.I., Hollis B.W., Howard N.S., Ascherio A. (2006). Serum 25-Hydroxyvitamin D Levels and Risk of Multiple Sclerosis. J. Am. Med. Assoc..

[221] Thouvenot E., Orsini M., Daures J.P., Camu W. (2015). Vitamin D Is Associated with Degree of Disability in Patients with Fully Ambulatory Relapsing-Remitting Multiple Sclerosis. Eur. J. Neurol..

[222] Oliveira S.R., Simão A.N.C., Alfieri D.F., Flauzino T., Kallaur A.P., Mezzaroba L., Lozovoy M.A.B., Sabino B.S., Ferreira K.P.Z., Pereira W.L.C.J. (2017). Vitamin D Deficiency Is Associated with Disability and Disease Progression in Multiple Sclerosis Patients Independently of Oxidative and Nitrosative Stress. J. Neurol. Sci..

[223] Smolders J., Menheere P., Kessels A., Damoiseaux J., Hupperts R. (2008). Association of Vitamin D Metabolite Levels with Relapse Rate and Disability in Multiple Sclerosis. Mult. Scler..

[224] Zhang Y., Liu G., Han X., Dong H., Geng J. (2016). The Association of Serum 25-Hydroxyvitamin D Levels with Multiple Sclerosis Severity and Progression in a Case-Control Study from China. J. Neuroimmunol..

[225] Mandia D., Ferraro O.E., Nosari G., Montomoli C., Zardini E., Bergamaschi R. (2014). Environmental Factors and Multiple Sclerosis Severity: A Descriptive Study. Int. J. Environ. Res. Public Health.

[226] Ascherio A., Munger K.L., White R., Köchert K., Simon K.C., Polman C.H., Freedman M.S., Hartung H.P., Miller D.H., Montalbán X. (2014). Vitamin D as an Early Predictor of Multiple Sclerosis Activity and Progression. JAMA Neurol..

[227] Muris A.H., Smolders J., Rolf L., Klinkenberg L.J.J., van der Linden N., Meex S., Damoiseaux J., Hupperts R. (2016). Vitamin D Status Does Not Affect Disability Progression of Patients with Multiple Sclerosis over Three Year Follow-Up. PLoS ONE.

[228] Fitzgerald K.C., Munger K.L., Köchert K., Arnason B.G.W., Comi G., Cook S., Goodin D.S., Filippi M., Hartung H.P., Jeffery D.R. (2015). Association of Vitamin D Levels with Multiple Sclerosis Activity and Progression in Patients Receiving Interferon Beta-1b. JAMA Neurol..

[229] Muris A.H., Rolf L., Broen K., Hupperts R., Damoiseaux J., Smolders J. (2016). A Low Vitamin D Status at Diagnosis Is Associated with an Early Conversion to Secondary Progressive Multiple Sclerosis. J. Steroid Biochem. Mol. Biol..

[230] Correale J., Ysrraelit M.C., Gaitán M.I. (2011). Vitamin D-Mediated Immune Regulation in Multiple Sclerosis. J. Neurol. Sci..

[231] Soilu-Hänninen M., Laaksonen M., Laitinen I., Erälinna J.P., Lilius E.M., Mononen I. (2008). A Longitudinal Study of Serum 25-Hydroxyvitamin D and Intact Parathyroid Hormone Levels Indicate the Importance of Vitamin D and Calcium Homeostasis Regulation in Multiple Sclerosis. J. Neurol. Neurosurg. Psychiatry.

[232] Simpson S., Taylor B., Blizzard L., Ponsonby A.L., Pittas F., Tremlett H., Dwyer T., Gies P., van der Mei I. (2010). Higher 25-Hydroxyvitamin D Is Associated with Lower Relapse Risk in Multiple Sclerosis. Ann. Neurol..

[233] James E., Dobson R., Kuhle J., Baker D., Giovannoni G., Ramagopalan S.V. (2013). The Effect of Vitamin D-Related Interventions on Multiple Sclerosis Relapses: A Meta-Analysis. Mult. Scler..

[234] Mokry L.E., Ross S., Ahmad O.S., Forgetta V., Smith G.D., Goltzman D., Leong A., Greenwood C.M., Thanassoulis G., Richards J.B. (2015). Vitamin D and Risk of Multiple Sclerosis: A Mendelian Randomization Study. PLoS Med..

[235] Harroud A., Manousaki D., Butler-Laporte G., Mitchell R.E., Davey Smith G., Richards J.B., Baranzini S.E. (2021). The relative contributions of obesity, vitamin D, leptin, and adiponectin to multiple sclerosis risk: A Mendelian randomization mediation analysis. Mult. Scler..

[236] Vandebergh M., Dubois B., Goris A. (2022). Effects of Vitamin D and Body Mass Index on Disease Risk and Relapse Hazard in Multiple Sclerosis: A Mendelian Randomization Study. Neurol. Neuroimmunol. Neuroinflamm..

[237] Derakhshandi H., Etemadifar M., Feizi A., Abtahi S.H., Minagar A., Abtahi M.A., Abtahi Z.A., Dehghani A., Sajjadi S., Tabrizi N. (2013). Preventive Effect of Vitamin D3 Supplementation on Conversion of Optic Neuritis to Clinically Definite Multiple Sclerosis: A Double Blind, Randomized, Placebo-Controlled Pilot Clinical Trial. Acta Neurol. Belg..

[238] Achiron A., Givon U., Magalashvili D., Dolev M., Liraz Zaltzman S., Kalron A., Stern Y., Mazor Z., Ladkani D., Barak Y. (2015). Effect of Alfacalcidol on Multiple Sclerosis-Related Fatigue: A Randomized, Double-Blind Placebo-Controlled Study. Mult. Scler..

[239] Shaygannejad V., Janghorbani M., Ashtari F., Dehghan H. (2012). Effects of Adjunct Low-Dose Vitamin D on Relapsing-Remitting Multiple Sclerosis Progression: Preliminary Findings of a Randomized Placebo-Controlled Trial. Mult. Scler. Int..

[240] Soilu-Hänninen M., Åivo J., Lindström B.M., Elovaara I., Sumelahti M.L., Färkkilä M., Tienari P., Atula S., Sarasoja T., Herrala L. (2012). A Randomised, Double Blind, Placebo Controlled Trial with Vitamin D3 as an Add on Treatment to Interferon β-1b in Patients with Multiple Sclerosis. J. Neurol. Neurosurg. Psychiatry.

[241] Kampman M.T., Steffensen L.H., Mellgren S.I., Jørgensen L. (2012). Effect of Vitamin D3 Supplementation on Relapses, Disease Progression, and Measures of Function in Persons with Multiple Sclerosis: Exploratory Outcomes from a Double-Blind Randomised Controlled Trial. Mult. Scler..

[242] Sotirchos E.S., Bhargava P., Eckstein C., van Haren K., Baynes M., Ntranos A., Gocke A., Steinman L., Mowry E.M., Calabresi P.A. (2016). Safety and Immunologic Effects of High- vs Low-Dose Cholecalciferol in Multiple Sclerosis. Neurology.

[243] Golan D., Halhal B., Glass-Marmor L., Staun-Ram E., Rozenberg O., Lavi I., Dishon S., Barak M., Ish-Shalom S., Miller A. (2013). Vitamin D Supplementation for Patients with Multiple Sclerosis Treated with Interferon-Beta: A Randomized Controlled Trial Assessing the Effect on Flu-like Symptoms and Immunomodulatory Properties. BMC Neurol..

[244] Stein M.S., Liu Y., Gray O.M., Baker J.E., Kolbe S.C., Ditchfield M.R., Egan G.F., Mitchell P.J., Harrison L.C., Butzkueven H. (2011). A Randomized Trial of High-Dose Vitamin D2 in Relapsing-Remitting Multiple Sclerosis. Neurology.

[245] Salari M., Janghorbani M., Etemadifar M., Dehghani A., Razmjoo H., Naderian G. (2015). Effects of Vitamin D on Retinal Nerve Fiber Layer in Vitamin D Deficient Patients with Optic Neuritis: Preliminary Findings of a Randomized, Placebo-Controlled Trial. J. Res. Med. Sci..

[246] O’Connell K., Sulaimani J., Basdeo S.A., Kinsella K., Jordan S., Kenny O., Kelly S.B., Murphy D., Heffernan E., Killeen R.P. (2017). Effects of Vitamin D3 in Clinically Isolated Syndrome and Healthy Control Participants: A Double-Blind Randomised Controlled Trial. Mult. Scler. J. Exp. Transl. Clin..

[247] Hupperts R., Smolders J., Vieth R., Holmøy T., Marhardt K., Schluep M., Killestein J., Barkhof F., Beelke M., Grimaldi L.M.E. (2019). Randomized Trial of Daily High-Dose Vitamin D3 in Patients with RRMS Receiving Subcutaneous Interferon β-1a. Neurology.

[248] Camu W., Lehert P., Pierrot-Deseilligny C., Hautecoeur P., Besserve A., Deleglise A.S.J., Payet M., Thouvenot E., Souberbielle J.C. (2019). Cholecalciferol in Relapsing-Remitting MS: A Randomized Clinical Trial (CHOLINE). Neurol. Neuroimmunol. Neuroinflamm..

[249] McLaughlin L., Clarke L., Khalilidehkordi E., Butzkueven H., Taylor B., Broadley S.A. (2018). Vitamin D for the Treatment of Multiple Sclerosis: A Meta-Analysis. J. Neurol..

[250] Dörr J., Bäcker-Koduah P., Wernecke K.D., Becker E., Hoffmann F., Faiss J., Brockmeier B., Hoffmann O., Anvari K., Wuerfel J. (2020). High-Dose Vitamin D Supplementation in Multiple Sclerosis—Results from the Randomized EVIDIMS (Efficacy of Vitamin D Supplementation in Multiple Sclerosis) Trial. Mult. Scler. J. Exp. Transl. Clin..

[251] Bhargava P., Cassard S., Steele S.U., Azevedo C., Pelletier D., Sugar E.A., Waubant E., Mowry E.M. (2014). The Vitamin D to Ameliorate Multiple Sclerosis (VIDAMS) Trial: Study Design for a Multicenter, Randomized, Double-Blind Controlled Trial of Vitamin D in Multiple Sclerosis. Contemp. Clin. Trials.

[252] Moen S.M., Celius E.G., Sandvik L., Nordsletten L., Eriksen E.F., Holmoøy T. (2011). Low Bone Mass in Newly Diagnosed Multiple Sclerosis and Clinically Isolated Syndrome. Neurology.

[253] Moen S.M., Celius E.G., Nordsletten L., Holmøy T. (2011). Fractures and Falls in Patients with Newly Diagnosed Clinically Isolated Syndrome and Multiple Sclerosis. Acta Neurol. Scand. Suppl..

[254] Dobson R., Ramagopalan S., Giovannoni G. (2012). Risk of Fractures in Patients with Multiple Sclerosis: A Population-Based Cohort Study. Neurology.

[255] Smolders J., Torkildsen Ø., Camu W., Holmøy T. (2019). An Update on Vitamin D and Disease Activity in Multiple Sclerosis. CNS Drugs.

[256] Slominski A.T., Zmijewski M.A., Plonka P.M., Szaflarski J.P., Paus R. (2018). How UV Light Touches the Brain and Endocrine System Through Skin, and Why. Endocrinology.

[257] Mattiuzzi C., Lippi G. (2020). Updates on Migraine Epidemiology. Eur. J. Neurol..

[258] Bussone G., Usai S., Grazzi L., Rigamonti A., Solari A., D’Amico D. (2004). Disability and Quality of Life in Different Primary Headaches: Results from Italian Studies. Neurol. Sci..

[259] Michel P., Dartigues J.F., Lindoulsi A., Henry P. (1997). Loss of Productivity and Quality of Life in Migraine Sufferers among French Workers: Results from the GAZEL Cohort. Headache.

[260] Leonardi M., Raggi A. (2019). A Narrative Review on the Burden of Migraine: When the Burden Is the Impact on People’s Life. J. Headache Pain.

[261] Antonaci F., Nappi G., Galli F., Manzoni G.C., Calabresi P., Costa A. (2011). Migraine and Psychiatric Comorbidity: A Review of Clinical Findings. J. Headache Pain.

[262] Schwedt T.J. (2014). Chronic Migraine. BMJ.

[263] Starling A.J., Vargas B.B. (2015). A Narrative Review of Evidence-Based Preventive Options for Chronic Migraine. Curr. Pain Headache Rep..

[264] Mitsikostas D.D., Rapoport A.M. (2015). New Players in the Preventive Treatment of Migraine. BMC Med..

[265] Diener H.C., Charles A., Goadsby P.J., Holle D. (2015). New Therapeutic Approaches for the Prevention and Treatment of Migraine. Lancet Neurol..

[266] Prakash S., Rathore C., Makwana P., Dave A., Joshi H., Parekh H. (2017). Vitamin D Deficiency in Patients with Chronic Tension-Type Headache: A Case-Control Study. Headache.

[267] Buettner C., Burstein R. (2015). Association of Statin Use and Risk for Severe Headache or Migraine by Serum Vitamin D Status: A Cross-Sectional Population-Based Study. Cephalalgia.

[268] Celikbilek A., Gocmen A.Y., Zararsiz G., Tanik N., Ak H., Borekci E., Delibas N. (2014). Serum Levels of Vitamin D, Vitamin D-Binding Protein and Vitamin D Receptor in Migraine Patients from Central Anatolia Region. Int. J. Clin. Pract..

[269] Prakash S., Kumar M., Belani P., Susvirkar A., Ahuja S. (2013). Interrelationships between Chronic Tension-Type Headache, Musculoskeletal Pain, and Vitamin D Deficiency: Is Osteomalacia Responsible for Both Headache and Musculoskeletal Pain?. Ann. Indian Acad. Neurol..

[270] Virtanen J.K., Giniatullin R., Mäntyselkä P., Voutilainen S., Nurmi T., Mursu J., Kauhanen J., Tuomainen T.P. (2017). Low Serum 25-Hydroxyvitamin D Is Associated with Higher Risk of Frequent Headache in Middle-Aged and Older Men. Sci. Rep..

[271] Knutsen K.V., Brekke M., Gjelstad S., Lagerløv P. (2010). Vitamin D Status in Patients with Musculoskeletal Pain, Fatigue and Headache: A Cross-Sectional Descriptive Study in a Multi-Ethnic General Practice in Norway. Scand. J. Prim. Health Care.

[272] Iannacchero R., Costa A., Squillace A., Gallelli L., Cannistrà U., de Sarro G. (2015). P060. Vitamin D Deficiency in Episodic Migraine, Chronic Migraine and Medication-Overuse Headache Patients. J. Headache Pain.

[273] Rapisarda L., Mazza M.R., Tosto F., Gambardella A., Bono F., Sarica A. (2018). Relationship between Severity of Migraine and Vitamin D Deficiency: A Case-Control Study. Neurol. Sci..

[274] Donmez A., Orun E., Sonmez F.M. (2018). Vitamin D Status in Children with Headache: A Case-Control Study. Clin. Nutr. ESPEN.

[275] Togha M., Razeghi Jahromi S., Ghorbani Z., Martami F., Seifishahpar M. (2018). Serum Vitamin D Status in a Group of Migraine Patients Compared With Healthy Controls: A Case-Control Study. Headache.

[276] Song T.J., Chu M.K., Sohn J.H., Ahn H.Y., Lee S.H., Cho S.J. (2018). Effect of Vitamin D Deficiency on the Frequency of Headaches in Migraine. J. Clin. Neurol..

[277] Hancı F., Kabakuş N., Türay S., Bala K.A., Dilek M. (2020). The Role of Obesity and Vitamin D Deficiency in Primary Headaches in Childhood. Acta Neurol. Belg..

[278] Rebecchi V., Gallo D., Princiotta Cariddi L., Piantanida E., Tabaee Damavandi P., Carimati F., Gallazzi M., Clemenzi A., Banfi P., Candeloro E. (2021). Vitamin D, Chronic Migraine, and Extracranial Pain: Is There a Link? Data From an Observational Study. Front. Neurol..

[279] Kjãrgaard M., Eggen A.E., Mathiesen E.B., Jorde R. (2012). Association between Headache and Serum 25-Hydroxyvitamin D: The Tromsø Study: Tromsø 6. Headache.

[280] Zandifar A., Masjedi S.S., Banihashemi M., Asgari F., Manouchehri N., Ebrahimi H., Haghdoost F., Saadatnia M. (2014). Vitamin D Status in Migraine Patients: A Case-Control Study. Biomed. Res. Int..

[281] Liampas I., Siokas V., Brotis A., Dardiotis E. (2020). Vitamin D serum levels in patients with migraine: A meta-analysis. Rev. Neurol..

[282] Liampas I., Bourlios S., Siokas V., Aloizou A.M., Dervenis P., Nasios G., Bakirtzis C., Bogdanos D.P., Dardiotis E. (2022). Vitamin D and tension-type headache: Causal association or epiphenomenon?. Int. J. Neurosci..

[283] Cayir A., Turan M.I., Tan H. (2014). Effect of Vitamin D Therapy in Addition to Amitriptyline on Migraine Attacks in Pediatric Patients. Braz. J. Med. Biol. Res..

[284] Mottaghi T., Askari G., Khorvash F., Maracy M.R. (2015). Effect of Vitamin D Supplementation on Symptoms and C-Reactive Protein in Migraine Patients. J. Res. Med. Sci..

[285] Buettner C., Nir R.R., Bertisch S.M., Bernstein C., Schain A., Mittleman M.A., Burstein R. (2015). Simvastatin and Vitamin D for Migraine Prevention: A Randomized, Controlled Trial. Ann. Neurol..

[286] Gazerani P., Fuglsang R., Pedersen J.G., Sørensen J., Kjeldsen J.L., Yassin H., Nedergaard B.S. (2019). A Randomized, Double-Blinded, Placebo-Controlled, Parallel Trial of Vitamin D 3 Supplementation in Adult Patients with Migraine. Curr. Med. Res. Opin..

[287] Edwards J.L., Vincent A.M., Cheng H.T., Feldman E.L. (2008). Diabetic Neuropathy: Mechanisms to Management. Pharmacol. Ther..

[288] Dyck P.J., Litchy W.J., Hokanson J.L., Low J.L., O’Brien P.C. (1995). Variables Influencing Neuropathic Endpoints: The Rochester Diabetic Neuropathy Study of Healthy Subjects. Neurology.

[289] Feldman E.L., Callaghan B.C., Pop-Busui R., Zochodne D.W., Wright D.E., Bennett D.L., Bril V., Russell J.W., Viswanathan V. (2019). Diabetic Neuropathy. Nat. Rev. Dis. Prim..

[290] Pop-Busui R., Boulton A.J.M., Feldman E.L., Bril V., Freeman R., Malik R.A., Sosenko J.M., Ziegler D. (2017). Diabetic Neuropathy: A Position Statement by the American Diabetes Association. Diabetes Care.

[291] Edmonds M., Manu C., Vas P. (2021). The Current Burden of Diabetic Foot Disease. J. Clin. Orthop. Trauma.

[292] Benbow S.J., Wallymahmed M.E., Macfarlane I.A. (1998). Diabetic Peripheral Neuropathy and Quality of Life. QJM.

[293] Soderstrom L.H., Johnson S.P., Diaz V.A., Mainous A.G. (2012). Association between Vitamin D and Diabetic Neuropathy in a Nationally Representative Sample: Results from 2001-2004 NHANES. Diabet Med..

[294] Shehab D., Al-Jarallah K., Mojiminiyi O.A., al Mohamedy H., Abdella N.A. (2012). Does Vitamin D Deficiency Play a Role in Peripheral Neuropathy in Type 2 Diabetes?. Diabet. Med.

[295] Chaychi L., Mackenzie T., Bilotta D., Lynch M., Cohen J. (2011). Association of Serum Vitamin D Level with Diabetic Polyneuropathy. Med. Pract. Rev..

[296] Fan L., Zhang Y., Zhu J., Song Y., Lin J. (2018). Association of Vitamin D Deficiency with Diabetic Peripheral Neuropathy and Diabetic Nephropathy in Tianjin, China. Asia Pac. J. Clin. Nutr..

[297] He R., Hu Y., Zeng H., Zhao J., Zhao J., Chai Y., Lu F., Liu F., Jia W. (2017). Vitamin D Deficiency Increases the Risk of Peripheral Neuropathy in Chinese Patients with Type 2 Diabetes. Diabetes Metab. Res. Rev..

[298] Yammine K., Abi Kharma J., Kaypekian T., Assi C., Zeeni N. (2022). Is Diabetic Neuropathy Associated with Vitamin D Status? A Meta-Analysis. Br. J. Nutr..

[299] McCarty M.F. (2005). Secondary Hyperparathyroidism Promotes the Acute Phase Response—A Rationale for Supplemental Vitamin D in Prevention of Vascular Events in the Elderly. Med. Hypotheses.

[300] Boulton A.J.M., Malik R.A. (1998). Diabetic Neuropathy. Med. Clin. N. Am..

[301] Faye P.A., Poumeaud F., Miressi F., Lia A.S., Demiot C., Magy L., Favreau F., Sturtz F.G. (2019). Focus on 1,25-Dihydroxyvitamin D3 in the Peripheral Nervous System. Front. Neurosci..

[302] Shillo P., Selvarajah D., Greig M., Gandhi R., Rao G., Wilkinson I.D., Anand P., Tesfaye S. (2019). Reduced Vitamin D Levels in Painful Diabetic Peripheral Neuropathy. Diabet. Med..

[303] Maser R.E., Lenhard M.J., Pohlig R.T. (2015). Vitamin D Insufficiency Is Associated with Reduced Parasympathetic Nerve Fiber Function in Type 2 Diabetes. Endocr. Pract..

[304] Pinzon R.T., Wijaya V.O., Veronica V. (2021). The Benefits of Add-on Therapy of Vitamin D 5000 IU to the Vitamin D Levels and Symptoms in Diabetic Neuropathy Patients: A Randomized Clinical Trial. J. Pain Res..

[305] Ghadiri-Anari A., Mozafari Z., Gholami S., Khodaei S.A., Aboutorabi-zarchi M., Sepehri F., Nadjarzade A., Rahmanian M., Namiranian N. (2019). Dose Vitamin D Supplementations Improve Peripheral Diabetic Neuropathy? A before-after Clinical Trial. Diabetes Metab. Syndr..

[306] Shehab D., Al-Jarallah K., Abdella N., Mojiminiyi O.A., al Mohamedy H. (2015). Prospective Evaluation of the Effect of Short-Term Oral Vitamin d Supplementation on Peripheral Neuropathy in Type 2 Diabetes Mellitus. Med. Princ. Pract..

[307] Basit A., Basit K.A., Fawwad A., Shaheen F., Fatima N., Petropoulos I.N., Alam U., Malik R.A. (2016). Vitamin D for the Treatment of Painful Diabetic Neuropathy. BMJ Open Diabetes Res. Care.

[308] Lee P., Chen R. (2008). Vitamin D as an Analgesic for Patients with Type 2 Diabetes and Neuropathic Pain. Arch. Intern. Med..

[309] Putz Z., Tordai D., Hajdú N., Vági O.E., Kempler M., Békeffy M., Körei A.E., Istenes I., Horváth V., Stoian A.P. (2022). Vitamin D in the Prevention and Treatment of Diabetic Neuropathy. Clin. Ther..

[310] Wei W., Zhang Y., Chen R., Qiu X., Gao Y., Chen Q. (2020). The Efficacy of Vitamin D Supplementation on Painful Diabetic Neuropathy: Protocol for a Systematic Review and Meta-Analysis. Medicine.

[311] Karonova T., Stepanova A., Bystrova A., Jude E.B. (2020). High-Dose Vitamin D Supplementation Improves Microcirculation and Reduces Inflammation in Diabetic Neuropathy Patients. Nutrients.

[312] Gonnelli S., Caffarelli C., Giordano N., Nuti R. (2015). The Prevention of Fragility Fractures in Diabetic Patients. Aging Clin. Exp. Res..

[313] Rossom R.C., Espeland M.A., Manson J.E., Dysken M.W., Johnson K.C., Lane D.S., Leblanc E.S., Lederle F.A., Masaki K.H., Margolis K.L. (2012). Calcium and Vitamin D Supplementation and Cognitive Impairment in the Women’s Health Initiative. J. Am. Geriatr. Soc..

[314] Annweiler C., Herrmann F.R., Fantino B., Brugg B., Beauchet O. (2012). Effectiveness of the Combination of Memantine plus Vitamin D on Cognition in Patients with Alzheimer Disease: A Pre-Post Pilot Study. Cogn. Behav. Neurol..

[315] Scragg R., Stewart A.W., Waayer D., Lawes C.M.M., Toop L., Sluyter J., Murphy J., Khaw K.T., Camargo C.A. (2017). Effect of Monthly High-Dose Vitamin D Supplementation on Cardiovascular Disease in the Vitamin D Assessment Study: A Randomized Clinical Trial. JAMA Cardiol..

[316] Ghorbani Z., Togha M., Rafiee P., Ahmadi Z.S., Rasekh Magham R., Djalali M., Shahemi S., Martami F., Zareei M., Razeghi Jahromi S. (2020). Vitamin D3 Might Improve Headache Characteristics and Protect against Inflammation in Migraine: A Randomized Clinical Trial. Neurol. Sci..

[317] Fallah R., Sarraf Yazd S., Sohrevardi S.M. (2020). Efficacy of Topiramate Alone and Topiramate plus Vitamin D3 in the Prophylaxis of Pediatric Migraine: A Randomized Clinical Trial. Iran. J. Child Neurol..

